# The antidepressive mechanism of Longya Lilium combined with Fluoxetine in mice with depression-like behaviors

**DOI:** 10.1038/s41540-024-00329-5

**Published:** 2024-01-13

**Authors:** Huina Ma, Hehua Huang, Chenyu Li, Shasha Li, Juefang Gan, Chunrong Lian, Yanwu Ling

**Affiliations:** 1 Department of Health, Youjiang Medical University for Nationalities, Baise, 533000 P. R. China; 2 Department of Human Anatomy, Youjiang Medical University for Nationalities, Baise, 533000 P. R. China

**Keywords:** Neuroscience, Cell biology

## Abstract

Traditional Chinese medicine is one of the most commonly used complementary and alternative medicine therapies for depression. Integrated Chinese-western therapies have been extensively applied in numerous diseases due to their superior efficiency in individual treatment. We used the meta-analysis, network pharmacology, and bioinformatics studies to identify the putative role of Longya Lilium combined with Fluoxetine in depression. Depression-like behaviors were mimicked in mice after exposure to the chronic unpredictable mild stress (CUMS). The underlying potential mechanism of this combination therapy was further explored based on in vitro and in vivo experiments to analyze the expression of COX-2, PGE2, and IL-22, activation of microglial cells, and neuron viability and apoptosis in the hippocampus. The antidepressant effect was noted for the combination of Longya Lilium with Fluoxetine in mice compared to a single treatment. COX-2 was mainly expressed in hippocampal CA1 areas. Longya Lilium combined with Fluoxetine reduced the expression of COX-2 and thus alleviated depression-like behavior and neuroinflammation in mice. A decrease of COX-2 curtailed BV-2 microglial cell activation, inflammation, and neuron apoptosis by blunting the PGE2/IL-22 axis. Therefore, a combination of Longya Lilium with Fluoxetine inactivates the COX-2/PGE2/IL-22 axis, consequently relieving the neuroinflammatory response and the resultant depression.

## Introduction

Depression is a prevalent mental health disorder affecting millions worldwide^1^. Persistent sadness, hopelessness, and loss of interest in daily activities^2^ characterize it. Despite multiple treatment options, including antidepressants, psychotherapy, and brain stimulation techniques, many depressed patients fail to achieve remission or experience adverse effects of medication^3^. Therefore, there is an urgent need to develop more effective and tolerable therapies for the treatment of depression^4^. Despite advances in chemical and synthetic drugs used to treat depression, substantial side effects and high costs are frequently reported in the treatment of depression^5–7^. There is evidence of an association between alterations in the immune system and depression, suggesting a potential mechanism for reducing neuroinflammation in treating depression^8^. Recent studies have suggested that neuroinflammation, or activation of immune cells in the brain, is a factor involved in the pathophysiology of depression^9^. This has led to an investigation of the use of anti-inflammatory drugs as potential antidepressants^10^. Notably, the chronic unpredictable mild stress (CUMS) model, as a prime example, has been widely recognized to mimic depression-like disorders in which animals are exposed to continuous unpredictable micro stress for prolonged periods^11^. This model develops representations of depression, such as stress-induced aphasia and apathy^12^. Therefore, the present study used CUMS to model depression-like disorders in mice^13^.

Longtooth lily (Lilium brownii var. viridulum), belonging to the genus Liliaceae, is often used as a functional component in traditional Chinese medicine and is widely distributed in Hunan and Jiangxi provinces of China^14^. Clinical research on the combination of Longya Lily and fluoxetine in the treatment of depression has been reported^15^, Current basic research generally believes that Longya Lily may exert pharmacological effects by regulating metabolism^16^, and fluoxetine treats depression by regulating GABA secretion and other treatments^17^. However, the molecular mechanism by which fluoxetine combined with Longya Lily exerts antidepressant effects is largely unknown. This study, through technical means such as cell and animal experiments, for the first time clarified that fluoxetine combined with Longya Lily exerts a therapeutic effect on depression by regulating inflammatory response through the COX-2/PGE2/IL-22 axis.

Evidence has been presented by Du et al. for the antidepressant activity of Longtooth lily, which confers a synergistic effect with albendoside that can modulate metabolic signaling^18^. Notably, a systematic review highlighted the clinical application of Lily of the Valley in treating depression, which points to the importance of elucidating the pharmacological basis of the mechanism^19^. As previously reported, gentian can modulate multiple miRNAs in depressed patients, affecting GABAergic synaptic and neurotrophic signaling, curbing neurotransmitter deficits, and attenuating inflammatory responses^20^. Fluoxetine is a selective 5-hydroxytryptamine reuptake inhibitor (SSRI) indicated for stimulating neurogenesis^21^. SSRIs are the most widely prescribed drugs for treating anxiety disorders, including depression^22^. In addition, fluoxetine has also been reported to improve depression-like behavior in CUMS rats^23^. In addition to being an antidepressant, fluoxetine has other therapeutic effects such as anxiety disorders, bulimia nervosa and premature ejaculation^24^. However, some adverse effects of SSRIs have been noted, such as nausea, nervousness, insomnia, headache, sexual dysfunction and hair loss (20)^25^. Therefore, synergistic drug and treatment goals are needed to prevent adverse events^26^.

Significant upregulation of cyclooxygenase-2 (COX-2) expression has been previously observed in the hippocampus of fluoxetine-resistant rats suffering from depression^27^. Inhibition of COX-2 exerts a neuroprotective effect on the dentate gyrus region, as evidenced by inhibition of neuroinflammatory responses and neuronal apoptosis, which is critical for the pathophysiology of depression^28^. Importantly, prostaglandin E2 (PGE2) constitutes a downstream factor of COX-2^29^. PGE2 is a critical inflammatory mediator involved in the pathophysiology of depression, and its concentration was increased in the brain and serum of rats receiving CUMS^30^. Also, published data suggest that PGE2 promotes T cells’ interleukin 22 (IL-22) production through its receptors EP2 and EP4^31^. A recent study found a significant positive correlation between IL-22 levels and depression^32^. In this study, the authors investigated the antidepressant effects and underlying mechanisms of a combination therapy that included the traditional herbal plant lobelia and fluoxetine, a commonly used antidepressant. They explored how this combination therapy could reduce neuroinflammation and provide superior antidepressant effects with fewer adverse effects than either agent.

## Results

### Longya Lilium combined with fluoxetine has a more significant antidepressant effect and fewer adverse reactions

A total of 462 related documents were retrieved. After screening, 8 documents met the criteria and were finally included. All of them were randomized controlled studies. A total of 736 cases of depression were included, consisting of 365 cases in the fluoxetine group and 371 cases in the Longya Lilium + fluoxetine group. A schematic diagram of the literature screening is shown in Supplementary Fig. 1, and the baseline characteristics of the included studies are presented in Supplementary Table 1.

**Fig. 1:**
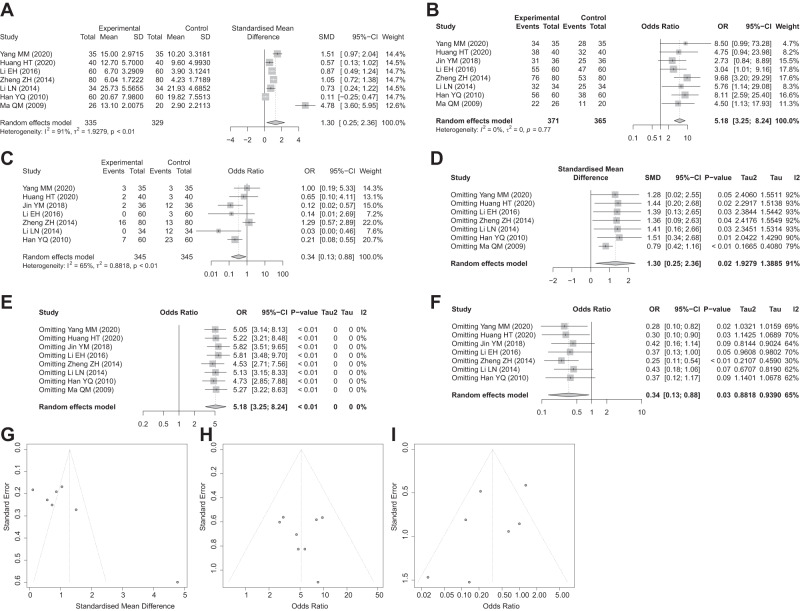
Main results of meta-analysis. **A** Forest plot comparing HAMD score between the fluoxetine group and the Longya Lilium + fluoxetine group. *I*^2^ = 91%, *p* < 0.01, with a random effect model used. **B** Forest plot comparing the total effective rate between the fluoxetine group and the Longya Lilium + fluoxetine group. *I*^2^ = 0%, *p* = 0.77, using the fixed-effect model. **C** Forest plot comparing adverse reaction rates between the fluoxetine group and the Longya Lilium + fluoxetine group. *I*^2^ = 65%, *p* < 0.01, with a random effect model used. **D** Sensitivity analysis results of HAMD score. **E** Sensitivity analysis results of total effective rate. **F** Sensitivity analysis results of adverse reaction rate. **G** Begg test results of HAMD score. **H** Begg test results of total effective rate. **I** Begg test results of adverse reaction rate.

Heterogeneity test results show that based on Hamilton Depression Scale (HAMD) score, there was heterogeneity among the studies (*I*^*2*^ = 91% and *P*_*h*_ < 0.01), and the random effect model was used. In terms of total effective rate, due to no heterogeneity among studies (*I*^*2*^ = 0% and *P*_*h*_ = 0.77), the fixed-effect model was adopted. In terms of the incidence of adverse reactions, there was heterogeneity among studies (*I*^*2*^ = 65.0% and *P*_*h*_ < 0.01), and the random effect model was conducted. The main results of the meta-analysis showed that compared with the fluoxetine group, the HAMD score in the fluoxetine + Longya Lilium group was decreased more significantly (SMD = 1.30, 95%CI = 0.25–2.36), the total effective rate was higher (OR = 5.18, 95%CI = 3.25–8.24), and adverse reactions were fewer (OR = 0.34, 95%CI = 0.13– 0.88) (Fig. 1A–C).

Meta-regression results indicated that the three indicators of HAMD score (*p* = 0.834), total effective rate (*p* = 0.747), and adverse reactions (*p* = 0.364) were all *p* > 0.05, suggesting that the publication period may not be related to the heterogeneity between studies. Further sensitivity analysis presented that the SMD or OR value did not change much, which demonstrated that the results of the Meta-analysis were reliable (Fig. 1D–F). In addition, Egger test results showed that the three indicators of HAMD score, total effective rate, and adverse reactions were all scattered in the funnel, indicating a reduced risk of publication bias and strong credibility of the article (Fig. 1G–I).

In summary, compared with treatment with fluoxetine alone, the antidepressant effect of Longya Lilium combined with fluoxetine may be more significant, accompanied by fewer adverse reactions.

### Longya Lilium, combined with fluoxetine, exerts an antidepressant therapeutic effect by downregulating the expression of COX-2

**Fig. 2:**
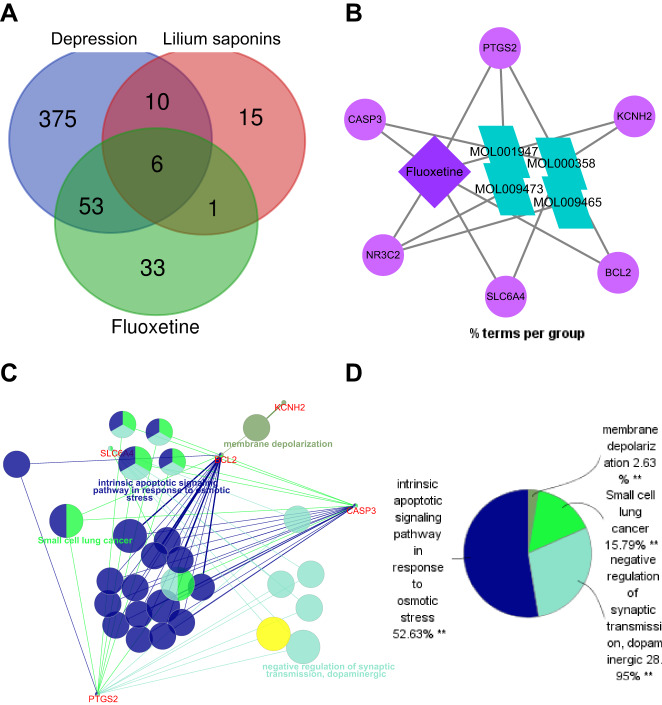
Drug target screening and gene function analysis of network pharmacology. **A** Venn diagram of fluoxetine-, Longya Lilium- and depression-related genes retrieved from the CTD and TCMSP databases. **B** fluoxetine-Longya Lilium-target regulatory network constructed by the Cytoscape software. **C**, **D** Functional enrichment analysis on the 6 critical genes by the ClueGO plug-in in Cytoscape software. Panel **C** is the network diagram of six essential genes and function enrichment, and **D** is the pie chart of function enrichment of six essential genes, where different colors indicate different categories of functional clusters. **p < 0.01, indicating the significance of the gene functional enrichment analysis results.

Next, the key targets of Longya Lilium combined with fluoxetine in the occurrence of depression through the network pharmacology database. Following retrieval of the CTD and TCMSP databases, 93 fluoxetine-related target genes, 447 depression-related target genes, and 33 saponin-related targets were obtained, respectively. Venn diagram analysis of these predicted targets showed NR3C2, SLC6A4, PTGS2 (COX-2), BCL2, KCNH2 and CASP3 at the intersection (Fig. 2A, Supplementary Table 2).

Figure 2B illustrates the network of Longya Lilium-fluoxetine-target constructed by Cytoscape software, and further functional enrichment analysis using the ClueGO plug-in suggested that the 6 genes were mainly enriched in intrinsic apoptotic signaling pathway in response to osmotic stress (GO:0008627), negative regulation of synaptic transmission, dopaminergic (GO:0032227) and Small cell lung cancer (KEGG:05222), of which COX-2 was at the core, and also enriched in the above functions (Fig. 2C, D). Meanwhile, published literature has shown that the mRNA expression of COX-2 in the peripheral blood of patients with depression was significantly higher than that of healthy individuals^33^. Both Lily and fluoxetine can inhibit the expression of COX-2^34,35^. Therefore, we selected COX-2 as the vital candidate gene.

These findings suggested that Longya Lilium combined with fluoxetine regulated the expression of COX-2 to exert the antidepressant therapeutic effect.

### Longya Lilium combined with fluoxetine significantly improves depression-like behaviors in mice

**Fig. 3:**
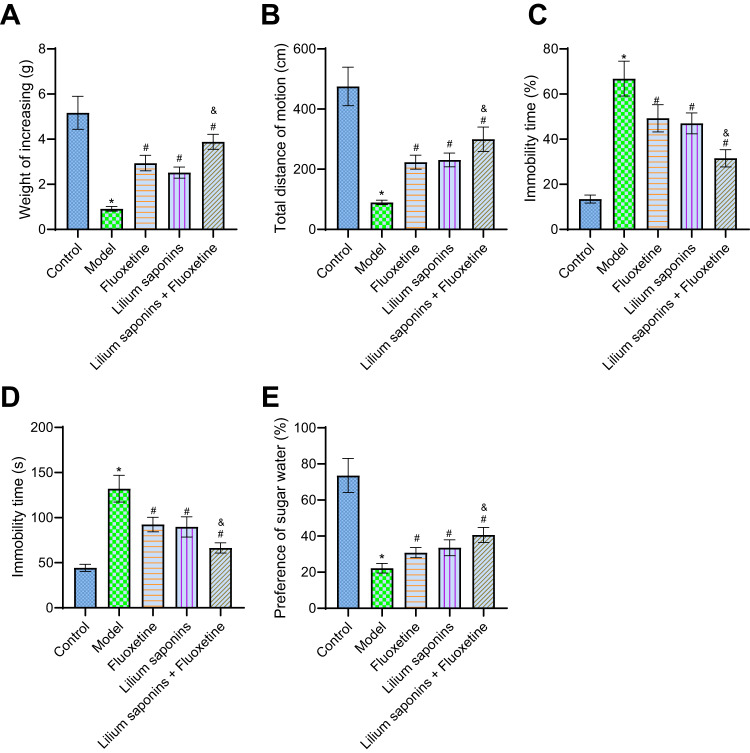
Longya Lilium combined with fluoxetine improves depression-like behaviors in mice. **A** The Body weight of mice in response to Longya Lilium alone or combined with fluoxetine. **B** Total distance of motion of mice in response to Longya Lilium alone or combined with fluoxetine. **C** Immobility duration of swimming of mice in response to Longya Lilium alone or combined with fluoxetine. **D** Immobility duration of tail suspension of mice in response to Longya Lilium alone or combined with fluoxetine. **E** Sucrose preference test of mice in response to Longya Lilium alone or combined with fluoxetine. Measurement data were expressed as mean ± standard deviation. One-way ANOVA with Tukey’s post-hoc test was used for comparison between multiple groups. *n* = 10 for mice per group. *p < 0.05, compared with the control mice. #*p* < 0.05, compared with the CUMS mice. &*p* < 0.05, compared with the fluoxetine-treated CUMS mice.

To elucidate the antidepressant effect of Longya Lilium combined with fluoxetine, we used the CUMS method to construct a mouse model of depression and carried out weight statistics, OFT, FST, TST, and sucrose preference tests. The obtained results showed that on the 28th day of the experiment, the CUMS mice exhibited a decline in body weight, the total distance of motion, and sucrose preference, yet an increase in the immobility duration of swimming and tail suspension (Fig. 3A–E). Treatment with fluoxetine, Longya Lilium and Longya Lilium + fluoxetine significantly improved the depression in modeled mice. Relative to fluoxetine or Longya Lilium treatment alone, combined treatment with Longya Lilium and fluoxetine presented noticeable improvement (Fig. 3A–E). The above results showed that the antidepressant effect of the combination of Longya Lilium and fluoxetine was more excellent than that of individual Longya Lilium or fluoxetine.

### Longya Lilium combined with fluoxetine alleviates depression and reduces neuroinflammatory response by inhibiting the expression of COX-2

**Fig. 4:**
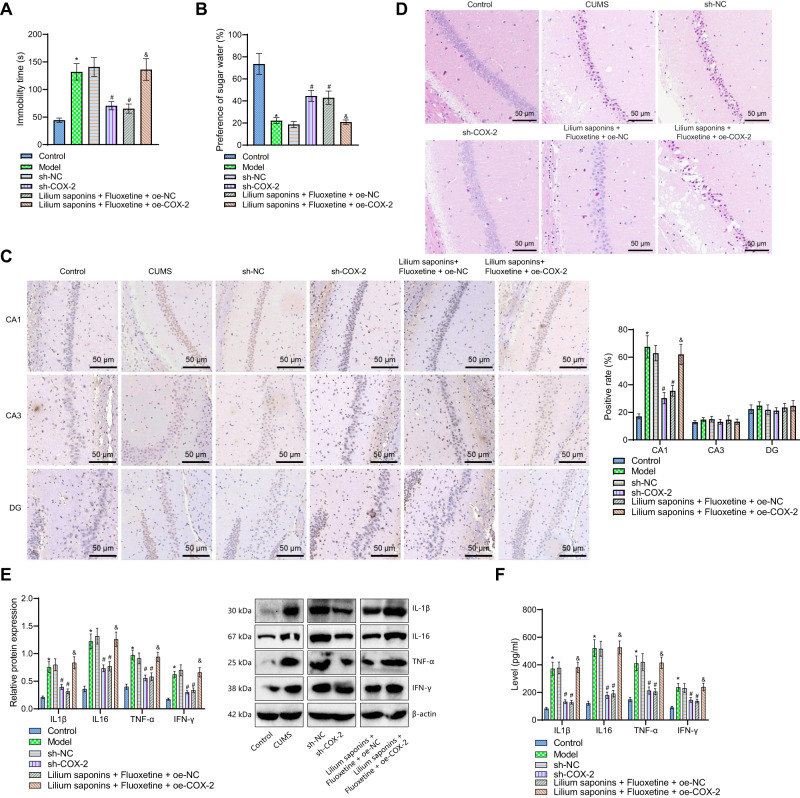
Longya Lilium combined with fluoxetine attenuates depression by inhibiting the expression of COX-2. **A** Immobility duration of tail suspension of mice in response to Longya Lilium + fluoxetine, sh-COX-2, or oe-COX2. **B** Sucrose preference test of mice in response to Longya Lilium + fluoxetine, sh-COX-2, or oe-COX2. **C** Positive expression of COX-2 protein in hippocampal CA1, CA3, and DG regions of mice in response to Longya Lilium + fluoxetine, sh-COX-2, or oe-COX2 detected by immunohistochemistry. **D** HE staining analysis of hippocampal CA1 area of mice in response to Longya Lilium + fluoxetine, sh-COX-2, or oe-COX2. **E** Western blot analysis of IL1β, IL-6, TNF-α, and IFN-γ proteins in hippocampal CA1 area of mice in response to Longya Lilium + fluoxetine, sh-COX-2, or oe-COX2. **F** Levels of IL1β, IL-6, TNF-α, and IFN-γ in the peripheral blood of mice in response to Longya Lilium + fluoxetine, sh-COX-2, or oe-COX2 measured by ELISA. Measurement data were expressed as mean ± standard deviation. One-way ANOVA with Tukey’s post-hoc test was used for comparison between multiple groups. *n* = 10 for mice per group. *p < 0.05, compared with the control mice. #*p* < 0.05, compared with the sh-NC-treated CUMS mice. &*p* < 0.05, compared with the Longya Lilium + fluoxetine + oe-NC-treated CUMS mice.

We then aimed to determine the mechanism of Longya Lilium combined with fluoxetine reducing depression and neuroinflammatory response. The results of TST and sucrose preference test showed an increase in the immobility duration of tail suspension and a decrease in sucrose preference in CUMS mice, while an opposite result was noted in the presence of sh-COX-2 or Longya Lilium + fluoxetine + oe-NC. In addition, treatment with Longya Lilium + fluoxetine + oe-COX-2 led to increased immobility duration of the tail suspension and decreased sucrose preference (Fig. 4A, B).

The immunohistochemistry results demonstrated that the positive expression of COX-2 was increased in the hippocampal CA1 region of CUMS mice and mainly present in the pyramidal cells. Treatment with sh-COX-2 or Longya Lilium + fluoxetine + oe-NC reduced the COX-2 positive expression, which was negated in response to Longya Lilium + fluoxetine + oe-COX-2. In the CA3 and DG regions, there was no difference in the positive expression of COX-2 upon each treatment (Fig. 4C).

In addition, HE staining data exhibited that control mice had more cells in the hippocampus, with compact cell hierarchy, standard size, complete structure, few amoebocytes, and dark stained nuclei. Conversely, the CUMS mice showed incomplete hippocampal cell morphology, nucleus pyknosis, enlarged intercellular space, and disordered arrangement. In the presence of sh-COX-2 or Longya Lilium + fluoxetine + oe-NC, nerve cell morphology was relatively complete, the staining and the layers between cells were clear, and amoebocytes were reduced. In contrast, further COX-2 overexpression resulted in more messy and loose cell arrangement, incomplete cell morphology, more amoebocytes and light staining (Fig. 4D).

In addition, Western blot analysis showed that expression of inflammatory factors IL1β, IL-6, TNF-α, and IFN-γ in the hippocampal CA1 region of CUMS mice increased as that of control mice. Following treatment with sh-COX-2 or Longya Lilium + fluoxetine + oe-NC, the expression of these factors was reduced compared with control mice, while the expression of these factors was elevated after further COX-2 overexpression (Fig. 4E). Moreover, ELISA data also revealed higher levels of inflammatory factors IL1β, IL-6, TNF-α, and IFN-γ in the peripheral blood of CUMS mice than those of control mice. The levels of these inflammatory factors were decreased in the peripheral blood of CUMS mice treated with sh-COX-2 or Longya Lilium + fluoxetine + oe-NC relative to CUMS mice, while these factors were increased following additional overexpression of COX-2 in the peripheral blood of CUMS mice (Fig. 4F).

In summary, overexpression of COX-2 may partially reverse the antidepressant effect of Longya Lilium combined with fluoxetine through its pro-inflammatory effect.

### COX-2 activates microglial cells and promotes inflammation through the PGE2/IL-22 axis

Neuroinflammatory response is a significant risk factor in the pathophysiology of depression. Research has shown that COX-2 promotes the production of pro-inflammatory prostaglandin E2 (PGE2), and enhanced PGE2 pathway activity effectively induces the core symptoms of depression^36^. Previous studies have indicated that PGE2 is an essential downstream factor of COX-2^37^ and plays a crucial role in the neuroprotective effects of SY5Y cells^38^. Therefore, it is hypothesized that Dragon Tooth Lily and Fluoxetine may affect depression through the COX-2/PGE2 pathway. In addition, IL-22 is an essential inflammatory factor regulated by PGE2 in immune cells^39^, and PGE2 promotes IL-22 production in T cells, thereby contributing to the development of allergic contact dermatitis^40^. Moreover, levels of IL-22 are positively associated with depression, and inhibiting IL-22 expression improves depressive behavior in mice models of colorectal cancer comorbid with depression^41,42^. Furthermore, analysis of the scRNASeqDB database reveals that COX-2 is mainly distributed in cortex microglia and hippocampus microglia, while PGE2 and IL-22 are predominantly found in endothelial cells and Oligodendrocyte progenitor cells (OPC), with microglia also exhibiting specific distribution patterns (Supplementary Fig. 2). Therefore, it is speculated that COX-2 may participate in the inflammatory response and contribute to the progression of depression through the PGE2/IL-22 axis, activating microglia in particular.

**Fig. 5:**
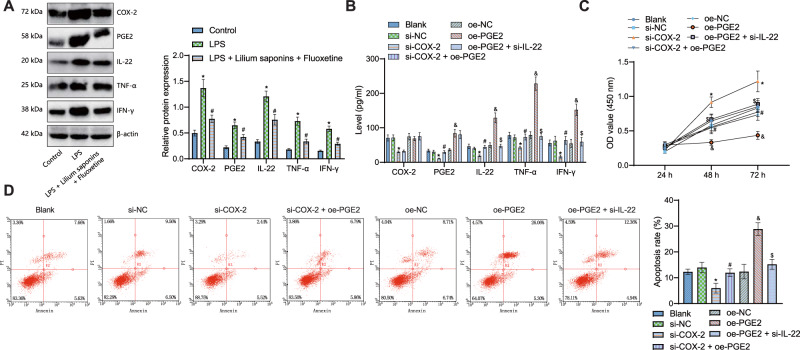
COX-2 activated microglial cells and consequently induces inflammation by activating the PGE2/IL-22 axis. **A** Western blot analysis of COX-2, PGE2, IL-22, TNF-α, and IFN-γ proteins in LPS-induced cells treated with Longya Lilium + fluoxetine. LPS-induced cells were treated with si-COX-2, si-COX-2 + oe-PGE2, oe-PGE2 or oe-PGE2 + si-IL-22. **B** COX-2, PGE2, IL-22, TNF-α, and IFN-γ expression in the supernatant of LPS-induced cells measured by ELISA. **C** Cell viability measured by MTT assay. **D** Cell apoptosis measured by flow cytometry. Measurement data were expressed as mean ± standard deviation. One-way or two-way ANOVA with Tukey’s post-hoc test was used to compare multiple groups. The cell experiment was repeated three times independently. *p < 0.05, compared with the si-NC-treated cells. #*p* < 0.05, compared with the si-COX-2-treated cells. &*p* < 0.05, compared with the oe-NC-treated cells. $*p* < 0.05, compared with the oe-PGE2-treated cells.

To further explore whether COX-2-activated microglia-mediated inflammatory response is involved in depression through the PGE2/IL-22 axis, LPS-induced BV-2 cell activation was used to simulate the in vitro neuroinflammatory response model. Western blot analysis data showed that protein expression of COX-2, PGE2, IL-22, TNF-α, and IFN-γ in the LPS cells was higher than in the control cells. In comparison to LPS-induced BV-2 cells, protein expression of COX-2, PGE2, IL-22, TNF-α, and IFN-γ was reduced in LPS-induced BV-2 cells treated with Longya Lilium and fluoxetine (Fig. 5A).

Moreover, ELISA results showed that silencing of COX-2 reduced the expression of COX-2, PGE2, IL-22, TNF-α, and IFN-γ in LPS-induced BV-2 cells. By comparison with LPS-induced BV-2 cells transduced with si-COX-2, the expression of COX-2, PGE2, IL-22, TNF-α, and IFN-γ in LPS-induced BV-2 cells was elevated in LPS-induced BV-2 cells transduced with si-COX-2 + oe-PGE. Overexpression of PGE2 elevated expression of COX-2, PGE2, IL-22, TNF-α, and IFN-γin LPS-induced BV-2 cells, but the expression of COX-2, PGE2, IL-22, TNF-α, and IFN-γ was reduced in LPS-induced BV-2 cells following additional silencing of IL-22 relative to overexpression of PGE2 alone (Fig. 5B).

The results of MTT and flow cytometry suggested that the viability of the si-COX-2-treated cells was enhanced, but apoptosis was reduced, which was negated by dual treatment with si-COX-2 and oe-PGE2. In addition, cell viability was attenuated, and apoptosis was enhanced in the presence of overexpression of PGE2, the effect of which was abolished by further silencing of IL-22 (Fig. 5C, D).

The above results suggested that COX-2 activated microglial cells to promote inflammation by activating the PGE2/IL-22 axis.

### Longya Lilium combined with fluoxetine inhibits neuroinflammatory response in mice with depression by suppressing the COX-2/PGE2/IL-22 axis

**Fig. 6:**
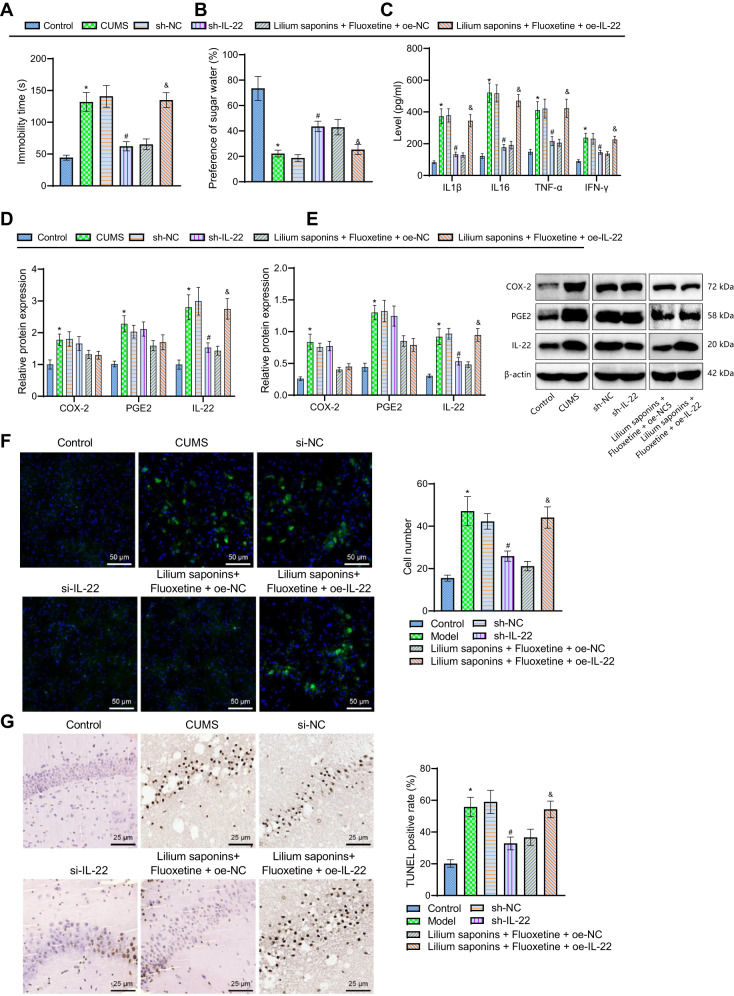
Longya Lilium, combined with fluoxetine, arrests neuroinflammatory response and depression-like behaviors in mice by inhibiting the COX-2/PGE2/IL-22 axis. CUMS mice were treated with si-IL-22 or Longya Lilium + fluoxetine + oe-IL-22. **A** Immobility duration of tail suspension of CUMS mice. **B** Sucrose preference test of CUMS mice. **C** Levels of IL1β, IL-6, TNF-α, and IFN-γ in the peripheral blood of CUMS mice measured by ELISA. **D** Expression of COX-2, PGE2 and IL-22 in the hippocampal CA1 region of CUMS mice. **E** Western blot analysis of COX-2, PGE2 and IL-22 proteins in the hippocampal CA1 region of CUMS mice. **F** Immunofluorescence staining analysis of Iba1 protein in the hippocampal CA1 region of CUMS mice. **G** TUNEL-positive cells in the hippocampal CA1 region of CUMS mice. Measurement data were expressed as mean ± standard deviation. One-way ANOVA with Tukey’s post-hoc test was used for comparison between multiple groups. *n* = 10 for mice per group. *p < 0.05, compared with the control mice. #*p* < 0.05, compared with the sh-NC-treated CUMS mice. &*p* < 0.05, compared with the Longya Lilium + fluoxetine + oe-NC-treated CUMS mice.

Finally, we sought to identify whether Longya Lilium combined with fluoxetine can improve the neuroinflammatory response in mice with depression by inhibiting the COX-2/PGE2/IL-22 axis. The results of the TST and sucrose preference test demonstrated an increase in the immobility duration of tail suspension of CUMS mice and a decline in the sucrose preference. Conversely, silencing of IL-22 caused opposite results. In the presence of Longya Lilium + fluoxetine + oe-IL-22, the immobility duration of tail suspension was prolonged whereas the sucrose preference was weakened (Fig. 6A, B).

ELISA data suggested an enhancement in the levels of IL1β, IL-6, TNF-α, and IFN-γ in the peripheral blood of CUMS mice compared with control mice, while in comparison to CUMS mice, these levels reduced in the peripheral blood of CUMS mice treated with silencing of IL-22. In addition, treatment with Longya Lilium + fluoxetine + oe-IL-22 elevated the levels of IL1β, IL-6, TNF-α, and IFN-γ in the peripheral blood of CUMS mice relative to CUMS mice treated with Longya Lilium + fluoxetine + oe-NC (Fig. 6C).

RT-qPCR and Western blot analysis results indicated that expression of COX-2, PGE2, and IL-22 in the hippocampal CA1 region of CUMS mice was higher than that of control mice. Compared with CUMS mice, treatment with sh-IL-22, the expression of IL-22 reduced in the hippocampal CA1 region of CUMS mice, while there is a n-fold increase in IL-22 expression in the hippocampal CA1 region of CUMS mice treated with Longya Lilium + fluoxetine + oe-IL-22 relative to CUMS mice treated with Longya Lilium + fluoxetine + oe-NC (Fig. 6D, E).

Moreover, immunofluorescence staining results showed that the number of Iba1 positive cells was increased in the hippocampal CA1 region of CUMS mice, while it was decreased in the absence of IL-22. Compared with Longya Lilium + fluoxetine + oe-NC, more Iba1-positive cells were noted in the presence of Longya Lilium + fluoxetine + oe-IL-22 (Fig. 6F).

TUNEL staining data revealed an upward inclination in the number of apoptotic cells in the hippocampal CA1 region of CUMS mice but silencing of IL-22 reduced the apoptotic cells. Relative to treatment with Longya Lilium + fluoxetine + oe-NC, the number of apoptotic cells was higher in response to treatment with Longya Lilium + fluoxetine + oe-IL-22 (Fig. 6G).

In summary, silencing IL-22 could inhibit the inflammatory response and reduce depression-like behaviors in mice. At the same time, overexpression of IL-22 may partially abrogate the antidepressant effect of Longya Lilium combined with fluoxetine through its pro-inflammatory effect.

## Discussion

In this study, the literature screening process was employed to define the research question and scope, ensuring the inclusion of high-quality studies to guarantee accurate analysis results. In addition, it aimed to enhance the homogeneity of the research, enabling a more precise evaluation of result consistency and credibility while reducing bias risk and improving the trustworthiness of evidence, thereby making the research findings more practical.

It is essential to investigate the molecular mechanisms underlying the antidepressant effects of combination therapy with gentian and fluoxetine. The present study proves that combination therapy has superior therapeutic effects on depression-like behavior and neuroinflammation compared to either agent alone. However, the specific biological pathways involved are yet to be understood^43^ fully. Understanding the molecular mechanisms responsible for the effectiveness of this combination could provide insights to optimize this therapy^44^. Another reason is the urgent need to find alternative therapies to overcome the limitations of the current treatment for depression^45^. Molecular studies on the potential of Longya Lilium as a therapeutic option could open new areas of development for effective treatment modalities^46^. Finally, the potential application of this study goes beyond the treatment of depression, as it could provide essential insights into the underlying molecular mechanisms of natural compounds that improve mental health^47^. Thus, this investigation may be critical for developing new therapeutic options to improve the quality of life of depressed patients and advance mental health research.

Combined Chinese and Western medicine therapies have been widely used in human diseases due to their superior efficacy^48^. Our meta-analysis showed that gentian herb combined with fluoxetine had more pronounced antidepressant effects and fewer adverse effects^49^. Oral administration of Ziziphi spinosae lily powder suspension exhibited a specific antidepressant-like effect due to a reduction in tail suspension and swimming immobility time^50^. The results of a previous study showed that gentianoside, in combination with bailey saponin, had better antidepressant activity compared to bailey saponin or gentianoside alone in a rat CUMS model^51^. Fluoxetine, the first selective 5-hydroxytryptamine reuptake inhibitor, has been approved for the clinical treatment of depression because it improves depression-like behavior and monoamine neurotransmitter levels^52^. In addition, fluoxetine can effectively alleviate CUMS-induced depression-like behavior in mice by modulating the expression of lncRNAs in the hippocampus^53^. There is growing evidence that fluoxetine combination therapy has better antidepressant effects than treatment alone; for example, the standardized commercial ginseng extract G115® significantly enhanced the antidepressant-like effects of fluoxetine in FST^54,55^. Combined treatment with fluoxetine and 7,8-dihydroxyflavone significantly inhibited sucrose preference and depression-like behavior in FST, accompanied by a significant increase in autophagy, neuronal nuclei and Iba1 expression^56^.

In the current study, subsequent network pharmacological findings suggest that the antidepressant therapeutic effect of gentiana combined with fluoxetine is associated with inhibition of COX-2 expression^57^. Consistently, pharmacological inhibition of COX-2 showed promise in preventing increases in anxiety-like behaviors induced by acute stress^58^. COX-2 is associated with the inflammatory response, and its decreased levels indicate a marked suppression of the inflammatory response^59^. Neuroinflammation is a significant risk factor for the pathophysiology of depression^60^. reduced COX-2 expression in the hippocampus contributes to the amelioration of depression-like behaviors and hippocampal neuroinflammation induced by the stress of chronic social defeat^61^. Together, these data reveal the potential of COX-2 inhibition as a target for the treatment of depression because of its inhibitory effect on neuroinflammation^62^. The combination of gentian and fluoxetine represents a promising alternative to traditional monotherapy for depression^63^. The current investigation highlights the potential advantages of this therapeutic combination over either agent alone because of its ability to significantly reduce depression-like behavior and neuroinflammation in the preclinical setting^64^. This study also provides valuable insight into the underlying molecular mechanisms involved in this combination therapy^65^.

Previous research has demonstrated the significant role of PGE2 as a downstream factor of COX-2^37^. Moreover, it has been shown to play a crucial role in the neuroprotective effect observed in SY5Y cells^38^. Hence, we hypothesize that Longya Lily and fluoxetine may exert their effects through the COX-2/PGE2 pathway in depression. In addition, IL22 is a pivotal inflammatory factor regulated by PGE2 in immune cells^39^. Therefore, we selected the PGE2/IL22 axis as the downstream signaling pathway for this study. Specifically, this therapy appears to reduce COX-2 expression and inactivate the PGE2/IL-22 pathway, leading to reduced microglial activation, inflammation, and neuronal damage^66^. The importance of this combination therapy lies in its ability to provide more effective and tolerable treatment options for depressed patients and improve their quality of life^67^. In addition, the study’s focus on natural compounds, such as gentian, highlights the potential for alternative therapies derived from traditional medicinal sources^68^. The potential applications of such integrative therapies are numerous and reflect the growing need for novel and effective treatments in the mental health field^69^. Ultimately, this investigation represents an important step toward developing new treatment strategies for depression^70^.

Furthermore, further mechanistic investigations in the present study suggest that COX-2 activates microglia, thereby inducing neuroinflammation by activating the PGE2/IL-22 axis^66^. Indeed, COX-2 produces the pro-inflammatory factor PGE2 and the subsequent glial cell activation, leading to the core symptoms of depression in a rat model^71^. The synthesis of PGE2 is induced in the occurrence of neuroinflammation-associated depression^72^. Also, PGE2 mRNA expression has been elevated in CUMS-induced rat hippocampus and frontal cortex, and its inhibition can help ameliorate CUMS-induced depression-like behavior^73^. A previous study showed that PGE2 acts directly on type 3 innate lymphocytes and promotes their production IL-22^74^. Recently, paroxetine was reported to disrupt depression-like behavior by downregulating IL-22 expression in combination with chemotherapeutic agents^75^.

Interestingly, both lily and fluoxetine inhibited COX-2 expression^76^. Therefore, it can be concluded that the combination of Lobelia and fluoxetine significantly inhibited the neuroinflammatory response and ameliorated the resulting depression by inhibiting the COX-2/PGE2/IL-22 axis^77^. The COX-2/PGE2/IL-22 axis is an essential inflammatory pathway implicated in the pathogenesis of several neuropsychiatric disorders, including depression^78^. The current investigation provides essential insights into the role of this pathway in the antidepressant effects of gentian and fluoxetine combination therapy^79^. Studies have shown that this combination reduces COX-2 expression, thereby reducing microglia activation, inflammation and neuronal apoptosis through inactivation of the PGE2/IL-22 pathway^78^. This is important because the COX-2/PGE2/IL-22 axis is known to contribute to inflammation-associated neuronal damage and to promote the production of other pro-inflammatory mediators involved in the pathophysiology of depression. Therefore, understanding the molecular mechanisms underlying the activity of this inflammatory pathway in depression is crucial for the development of more effective treatments to reduce neuroinflammation and alleviate psychiatric symptoms^80^. The results of this study represent an essential contribution to this emerging field of research and may be vital in paving the way for novel therapeutic strategies targeting the COX-2/PGE2/IL-22 axis in depression and other neuropsychiatric disorders.

**Fig. 7:**
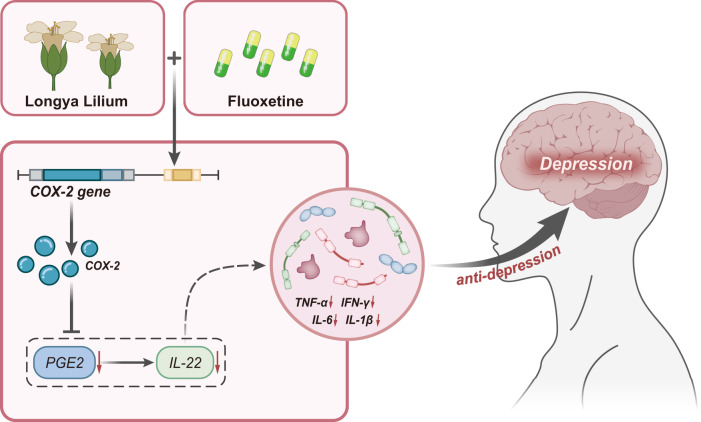
Schematic illustration of the mechanism of Longya Lilium combined with fluoxetine in depression. Longya Lilium combined with fluoxetine can inhibit the PGE2/IL-22 axis by inhibiting the expression of COX-2 and reducing neuroinflammation, thus exerting an antidepressant therapeutic effect.

Overall, the present study confirmed the antidepressant effect of the combination of gentian and fluoxetine. This effect may be related to the inhibition of the COX-2/PGE2/IL-22 axis (Fig. 7). The results of this study suggest that the combination of gentian herb and fluoxetine may be a potential alternative antidepressant treatment with fewer side effects than conventional monotherapy. Further studies are needed to elucidate the optimal dose and duration of this agent and examine the safety, tolerability, and efficacy of this combination in humans. Clinical trials could use a combination of behavioral and neuroimaging measures to understand better the effects of Longya Lilium and fluoxetine on the brain and clinical symptoms of depression. At the same time, the current study provides some insight into the underlying mechanisms of this combination therapy; the involvement of multiple pathways and components of the immune system warrants further investigation. In addition, studies evaluating the effects of Gentiana and fluoxetine in different populations, such as those with treatment-resistant or severe forms of depression, are needed to determine the potential efficacy of this combination in more challenging situations. However, these findings provide promising evidence for developing new treatment strategies to address the ongoing challenges in managing depression.

Although this study has explored in-depth the mechanisms of the combination of Longya Lily and Fluoxetine in the treatment of depression from multiple perspectives, there are still some limitations. Firstly, our study primarily focuses on the role of the COX-2/PGE2/IL-22 axis in neuroinflammatory response in depression, while investigating other possible inflammatory regulatory pathways is not comprehensive enough. Secondly, this study mainly observes the activation state of microglial cells and their associated inflammatory response, without delving into the role of astrocytes. Astrocytes are essential supportive cells in the central nervous system, and they may play a crucial role in the occurrence and development of neuroinflammation and depression. The lack of detailed investigation on astrocytes may result in an incomplete understanding of the overall neuroinflammatory regulatory mechanisms. In the future, we will further explore the role of Longya Lily and Fluoxetine in other neuroinflammatory regulatory pathways, especially in investigating the role of astrocytes in this process. Simultaneously, in-depth analysis of various biomarkers associated with depression and the effects of drugs on these biomarkers will provide a theoretical basis for individualized treatment and better outcomes for patients with depression.

## Methods

### Ethics statement

The current study was performed with the approval of the Ethics Committee of Youjiang Medical University for Nationalities and performed strictly with the Guide for the Care and Use of Laboratory Animals published by the US National Institutes of Health.

### Literature retrieval

From the inception to December 2020, PubMed, Embase, Web of Science, China National Knowledge Infrastructure (CNKI), and Wanfang databases were used to obtain relevant literature by retrieving depression, fluoxetine, Lilium, baihe, Lilii Bulbus; Lily Bulb, integrated Chinese and western medicine, and randomized controlled study. To screen the target documents accurately, we used the term NOT (review, animal experiment, meeting, Case report).

### Literature screening and data extraction

The inclusion criteria were: (1) publicly published documents at home and abroad; (2) randomized controlled study; (3) patients with depression according to the diagnostic criteria for depression; (4) intervention measures including Longya Lilium combined with fluoxetine. The exclusion criteria were: (1) duplicated publications; (2) studies without sufficient data; (3) the included patients had taken antidepressants before. Two reviewers independently extracted information from the selected studies. Any disputes regarding data extraction were resolved by agreement among several investigators.

### Meta-analysis

Meta-analysis was performed using R (v4.2.3), and the combined effect was evaluated using odds ratio (OR), weighted mean difference (WMD) and 95% confidence interval (CI). The Cochran Q test evaluated the heterogeneity test among studies. The *P*_*h*_ < 0.05 indicated that there is heterogeneity. *I*^2^ was used to evaluate the magnitude of heterogeneity. The *I*^2^ value ranged from 0 to 100%. The larger the *I*^2^ value was, the more pronounced the heterogeneity was. The *p* < 0.05 and *I*^2^ > 50% indicated significant heterogeneity among the studies, which the random effect model further analyzed. Otherwise, the fixed-effect model was used for analysis. Sensitivity analysis: the method of elimination one by one was used to evaluate the stability of the results. Stata software was used to conduct the Egger test to evaluate whether the included literature has publication bias and to clarify the reliability of the original analysis results.

### Drug and disease target screening of network pharmacology

Previous studies have shown that saponins are the main component responsible for the antidepressant effects of Bulbus Lilii, a traditional Chinese medicinal herb^81^. Hence, this study focuses on screening target genes directly related to saponins. Active ingredients and targets of Lilium saponins were retrieved from the Traditional Chinese Medicine Systems Pharmacology Database and Analysis Platform (TCMSP) database, and the targets related to the active ingredients of saponins were screened. The UniProtKB database retrieved the official gene symbol corresponding to the target, with the species set as “Homo sapiens”. Next, fluoxetine-related target genes were retrieved from the Comparative Toxicogenomics Database (CTD) database (Interaction Count ≥ 3). At the same time, the target genes related to depression were retrieved using the CTD database (Inference score ≥ 100). A Venn diagram of target genes related to Longya Lilium, fluoxetine, and depression was plotted using the Draw Venn Diagram tool.

### Drug-component-target network construction and enrichment analysis

Cytoscape 3.6.0 software was applied to construct the Longya Lilium-fluoxetine-gene regulatory network. ClueGO plug-in in Cytoscape software was used to perform function enrichment analysis to construct a gene-function-pathway regulatory relationship network. Different functions were clustered according to different colors, and a pie chart of gene function enrichment was drawn to display the main Kyoto Encyclopedia of Genes and Genomes (KEGG) and gene ontology (GO) functions.

### Visualization of single-cell sequencing data analysis results

The specific cell types involved in the regulation axis of COX-2/PGE2/IL-22 in brain tissue were retrieved from the scRNASeqDB database^82^, available at https://bioinfo.uth.edu/scrnaseqdb/. The distribution of COX-2 (PTGS2) in various types of brain tissue cells is shown in the following link: https://bioinfo.uth.edu/scrnaseqdb/index.php?r=site/geneView&id=PTGS2&set=GSE67835&csrt=9002072548059807797. Similarly, the distribution of PGE2 (PTGER2) in various types of brain tissue cells can be found at: https://bioinfo.uth.edu/scrnaseqdb/index.php?r=site/geneView&id=PTGER2&set=GSE67835&csrt=9002072548059807797. Lastly, the distribution of IL-22 (IL22) in different types of brain tissue cells can be viewed here: https://bioinfo.uth.edu/scrnaseqdb/index.php?r=site/geneView&id=IL22&set=GSE67835&csrt=9002072548059807797.

### Depression mouse model establishment

120 SPF C57BL/6J male mice (aged 9–12 weeks old, weighing 18–21 g) were used to develop a model of depression based on the CUMS method. The stress paradigm consisted of the following stressors: swimming in ice water (5 min), food and water deprivation (24 h for each), tail pinch (1 min), shaking (once/s, 15 min), reversal of day and night and bondage (5 min each time) for a total of 24 days. One of these stressors was randomly arranged every day, and each stimulus was performed 21 times so that the mice could not predict the occurrence of the stimulus. Ten mice were used as normal controls, and 110 were stimulated to establish a depression model. After 5 weeks, the behavioral test was performed, and the serum sample was collected for biochemical testing.

The overall grouping was as follows: Control group (normal mice, no treatment), Model group (model mice, intravenous injection of empty vector), Fluoxetine group (model mice, oral gavage of Fluoxetine at a dose of 20 mg/kg), Lilium saponins group (model mice, oral gavage of Lilium saponins at a dose of 50 mg/kg), Lilium saponins+Fluoxetine group (model mice, oral gavage of Lilium saponins at a dose of 25 mg/kg and Fluoxetine at a dose of 10 mg/kg). The gavage volume was 0.3 mL, administered once daily for a continuous period of 21 days. In this study, we used Lilium saponins (Shaanxi Feiste Biotechnology Co., Ltd., product code: 904) to treat the animals without using the whole or parts of Lilium brownii. This study employed lily saponins (Xi’an Feist Biotechnology Co., Ltd., product number 904) for animal treatment, without using whole or partial dragon tooth lilies. The concentration of Lilium saponins was determined based on the literature reference^83^, while the concentration of Fluoxetine was determined based on the literature reference^84^.

Animal experiments were conducted with the approval of our institution’s Animal Ethics Committee and in compliance with the guidelines for animal experimentation provided by the National Institutes of Health (NIH). Specifically, mice were anesthetized by intraperitoneal injection of sodium pentobarbital (60 mg/kg) while closely monitoring vital signs, including respiration, heart rate, and blood pressure, to ensure optimal anesthesia. When obtaining mouse brain tissue, humane euthanasia procedures were strictly followed, employing an overdose anesthesia method (2–4 times the typical anesthetic dosage) to ensure the mice’s gentle death. Prior to performing the procedures, it was ensured that the animal euthanasia equipment was in good working condition. Furthermore, operators underwent training to identify signs of pain, fear, or distress in experimental animals, avoiding any startling noises that could cause fear. After the procedure, operators were capable of determining mouse death in accordance with humane practices. Proper handling and disposal of animal remains were also ensured to prevent any environmental impact. All individuals involved in euthanasia procedures demonstrated sensitivity toward the value of animal life and adhered to scientific, rational, and ethical principles to ensure painless and fear-free death. It is essential to respect the dignity and worth of the experimental animals, rather than viewing them merely as research materials or tools^85–87^.

### Plasmid construction, lentivirus transfection, and grouping

BV-2 (ATL03001) purchased from National Infrastructure of Cell Line Resource (Beijing, China) were cultured in DMEM (SH30022.01B, Shanghai Suer Biotechnology Co., Ltd., Shanghai, China) supplemented with 10% FBS (SH30070.03, HyClone Laboratories, Logan, UT), 1% penicillin and streptomycin in a 5% CO_2_ incubator at 37 °C. Three siRNA sequences were designed for mouse-derived COX-2 CDS and IL-22 CDS and synthesized by Guangzhou RiboBio Co., Ltd. (Guangzhou, Guangdong, China). BV-2 cells were plated in a 24-well plate at 1 × 10^5^ cells/healthy density and cultured with 1 mL DMEM at 37 °C with 5% CO_2_ for 24 h. Lipofectamine 2000 reagent (#11668027, Invitrogen) was used to transduce 50 nM negative control (NC), COX-2 small interfering RNA (siRNA, si), or IL-22 siRNA into BV-2 cells for 48 h. The silencing efficiency of COX-2 and IL-22 in BV-2 cells was detected by Western blot.

The lentivirus packaging system was constructed by LV5-GFP (lentivirus gene overexpression vector) and pSIH1-H1-copGFP (lentivirus short hairpin RNA (sh RNA) fluorescent expression vector gene silencing vector). sh-COX-2, sh-IL-22, overexpression (oe)-COX-2, oe-IL-22, sh-NC, and oe-NC were completed by Shanghai GenePharma Co. Ltd. (Shanghai, China). BV-2 cells were co-transfected with packaged lentivirus and target vectors. After incubation for 48, cell supernatant was collected. The lentivirus particles in the supernatant after centrifugation were filtered to detect the lentivirus titer. G418 was used to screen the stably transfected cells for over 2 weeks, and the mRNA or protein levels were identified by reverse transcription-quantitative polymerase chain reaction (RT-qPCR) or Western blot analysis.

The CUMS-induced mice were intravenously injected with lentivirus (10 μL) harboring sh-NC, sh-COX-2, sh-IL-22, Longya Lilium + fluoxetine + oe-NC, Longya Lilium + fluoxetine + oe-COX-2 and Longya Lilium + fluoxetine + oe-IL-22 with a final titer of 1 × 10^9^ TU/mL. One day before CUMS, body weight, depression-like behaviors, and sucrose consumption were evaluated. On the second day, the mice underwent CUMS, and at the same time, were injected with lentiviruses and administrated with drugs *via* the tail vein. After 28 days, body weight, depression-like behaviors and sucrose consumption were assessed. The mice were anesthetized with sodium pentobarbital and euthanized; after that, the blood and brain tissue were collected, and the brain tissue was stored at −80 °C.

### Behavioral assessments

In this study, the body weight of mice was measured on the day before the experiment and again on the 28th day. The weight gain of each group of mice was calculated by subtracting the weight on the first day of the experiment from the weight on the 28th day.

An opaque cube measuring 80 cm × 80 cm × 40 cm was constructed for the open field test with 25 equal-sized squares marked on the floor. Mice were placed in the center square, and their activity was recorded for 3 min. Both horizontal and vertical activity were recorded, with horizontal activity being measured as the number of squares crossed and vertical activity as the number of times both forepaws left the ground. Each animal was tested only once, and to ensure the reliability of the experiment, a double-masked procedure was employed whereby different researchers monitored the activity without knowledge of the group assignment of the mice^88^.

In the forced swim test, mice were placed individually in a water tank (80 cm high, 30 cm in diameter, and at a temperature of 25 °C) and forced to swim for 6 min. After 24 h, the mice were placed in the same tank for 5 min, and their swimming and immobility times were recorded for 3 min. Immobility time was defined as floating with minimal movement to keep the head above water^36^.

In the tail suspension test, mice were subjected to a series of parameters while hanging by their tails for 360 s with their heads facing downwards, with the tail being fixed to support 1.5–2 cm from the base. The immobility time was recorded as the time the mouse spent motionless while in this state. The measurement was taken using a single-masked procedure^89^.

In the sucrose preference test, mice were individually housed in a cage containing two bottles of sucrose solution (1%, w/v) for 24 h during an adaptation stage. During the second 24-h period, one bottle of sucrose solution was replaced with tap water. In the test phase, mice were deprived of food and water for 24 h and then placed in a cage containing two bottles, one containing 100 mL of 1% sucrose solution and the other 100 mL of tap water, for 3 h. Sucrose preference was defined as the sucrose consumption divided by the total consumption (sucrose solution and water) multiplied by 100%. Eight mice were used for each group^36^.

### Hematoxylin-eosin (HE) staining

Paraffin-embedded sections of hippocampal tissues were prepared and cut into 4-μm-thick sections. The sections were stained with hematoxylin (C0007, Baoman Biotechnology Co., Ltd., Shanghai, China) for 10 min at room temperature. Next, the sections were counterstained with eosin at room temperature for 5–10 min. Before observation, the samples were mounted with neutral gum under an optical microscope (XSP-36, Boshida Optical Instrument Co., Ltd., Shenzhen, China).

### Immunohistochemistry

The tissue sections were incubated with 3% H_2_O_2_ (84885, Sigma-Aldrich, St Louis, MO) at 37 °C for 30 min and boiled in 0.01 M citrate buffer at 95 °C for 20 min. The sections were then blocked with normal goat serum at 37 °C for 10 min and probed with primary antibody rabbit anti-mouse COX-2 (1:100, ab15191) at 4 °C overnight. The following day, the sections were re-probed with secondary antibody goat anti-rabbit immunoglobulin (IgG; 1:1000, ab6721, Abcam Inc., Cambridge, UK) at room temperature for 30 min, developed with diaminobenzidine (DAB; ab64238, Abcam), counterstained with hematoxylin and mounted. The primary antibody was substituted with PBS as NC. Five high-power fields were randomly selected from each section, with 100 cells counted in each field, and the rate of positive cells was calculated.

### Enzyme-linked immunosorbent assay (ELISA)

Levels of IL-1β, IL-6, tumor necrosis factor-α (TNF-α) and interferon-gamma (IFN-γ) in peripheral blood of mice were measured using the IL-1β (ab197742, Abcam), IL-6 (ab222503, Abcam), TNF-α (ab208348, Abcam) and IFN-γ (ab100689, Abcam) ELISA kits. The optical density (OD) value was measured at 450 nm.

### Immunofluorescence staining

The mouse hippocampal tissue sections were fixed with 4% paraformaldehyde, permeabilized in 0.3% Triton X-100, and blocked with 1% bovine serum albumin. After that, the sections were probed with primary antibody Iba1 (A12391, 1:100, Abclonal Technology, Inc.) overnight at 4 °C. Following PBS washing, the sections were re-probed with corresponding fluorescent secondary antibodies against AS011 and AS007 (1:100, Abclonal). Finally, the sections were observed under a confocal laser scanning microscope (FluoView FV10i, Olympus Optical Co., Ltd, Tokyo, Japan). Five sections were randomly selected from each mouse, and three visual fields were randomly selected from each section for a photograph. ImageJ software (National Institutes of Health, Bethesda, Maryland) was used for fluorescence intensity analysis.

### TUNEL assay

TUNEL kit (Boehringer Mannheim, Germany) was used for TUNEL staining as the instructions described. The 20-µm-thick tissue cross-sections were prepared in a constant cold box cutter at −22 °C, and 10 sections were taken from each group for TUNEL staining. Under an optical microscope (Leica DM4 P, Shanghai Meijing Electronics Co., Ltd., Shanghai, China), the dark particles indicated apoptotic cells. Five high-power fields were randomly selected from each section for observation and photographing, and 100 positive stained cells were calculated.

### RNA isolation and quantitation

Total RNA was extracted with RNA Extraction Kit (D203-01, GenStar Biosolutions Co., Ltd., Beijing, China) and then reversely transcribed as per the instructions of TaqMan MicroRNA Assays Reverse Transcription Primer (4366596, Thermo Fisher Scientific Inc., Waltham, MA). RT-qPCR was conducted using SYBR^®^ Premix Ex Taq^TM^ II kit (RR820A, Action-award Biological Technology Co., Ltd, Guangzhou, China) on the ABI PRISM^®^ 7300 system (Prism^®^ 7300, Shanghai Kunke Instrument Equipment Co., Ltd., Shanghai, China). Takara Biotechnology Ltd. (Dalian, China) designed and synthesized the primers, with sequences shown in Supplementary Table 3. The fold changes were calculated using relative quantification (the 2^−ΔΔCt^ method), with GAPDH as a loading control.

### Western blot analysis

Total protein was extracted from tissues with tissue lysis buffer containing phenylmethylsulphonyl fluoride (PMSF) (Boster Biological Technology Co., Ltd., Wuhan, China). The concentration of the total protein was determined by a bicinchoninic acid (BCA) kit (20201ES76, YEASEN Biotechnology Co., Ltd., Shanghai, China). The protein was separated by SDS-PAGE using 10% separation gel and spacer gel and transferred onto the nitrocellulose membrane. The membrane was then blocked using 5% skimmed milk powder at 4 °C overnight and underwent overnight incubation at 4 °C with the diluted primary rabbit antibodies (Abcam) against COX-2 (1:500, ab62331, Abcam,), PGE2 (1:200, #PA5-77694, Thermo Fisher Scientific Inc., Waltham, MA), IL-22 (1:1000, #PA5-115408, Thermo Fisher Scientific), TNF-α (1:1000, ab215188, Abcam), and IFN-γ (1:100, ab24780). The next day, the immunocomplexes on the membrane were visualized using enhanced chemiluminescence (ECL) reagent (Pierce Biotechnology Inc., Rockford, IL) at room temperature for 1 min and band intensities were quantified using ImageJ software, with GAPDH serving as a loading control. All original western blot images can be found in Supplementary Figs. 3–25.

### Cell culture and grouping

BV-2 cells were cultured with high-glucose DMEM containing 10% FBS, 100 units/mL penicillin, and 100 μg/mL streptomycin in a 5% CO_2_ incubator at 37 °C, with the medium changed every 24 h. Cells were passaged after 48 h. After recovery, the growth status of BV-2 cells in the culture flask was observed under a microscope. When cells reached about 80–90% confluence, the medium was discarded, and 5 mL 0.01 M PBS was used to wash the culture flask twice to remove the residual medium. Next, 1 mL of 0.25% trypsin was added to the culture flask to cover the cells. The digestion was halted when the gap between the BV-2 cells under a microscope increased. The cells were gently pipetted until complete suspension and then passaged for another culture.

BV-2 cell suspension was seeded in a 96-well culture plate at a density of 1 × 10^4^ cells/well (100 μL) and incubated in a cell incubator for 12 h to allow cell adherence to the wall. Cells were treated with PBS and served as the control. After 1 h, the cells were administered 1 mg/L lipopolysaccharide (LPS) for 24 h and further treated with Longya Lilium (10 μM) + fluoxetine (15 μM), and lentivirus containing si-NC, si-COX-2, si-COX-2 + oe-PGE2, oe-NC, oe-PGE2, and oe-PGE2 + si-IL-22.

### MTT assay

Cells were seeded in a 96-well plate at a density of 5 × 10^4^ cells/well, with 6 parallel wells set in each group. After reoxygenation, 20 μL of MTT solution (Sigma-Aldrich) was added to the cells and incubated in a 37 °C incubator for 4 h. Each well was supplemented with 150 μL of methyl sulfoxide (Sigma-Aldrich. Afterward, each well’s optical density (OD) value was measured at 450 nm using a microplate reader.

### Flow cytometry

A flow cytometer assessed the cell apoptosis after 48 h of transfection. Following the instructions of Annexin-V-FITC Cell Apoptosis Detection Kit (CA1020, Beijing Solarbio Science & Technology Co., Ltd., Beijing, China), Annexin-V-FITC, PI, and HEPES buffer were prepared into Annexin-V-FITC/PI dye solution at a ratio of 1:2:50. A total of 1 × 10^6^ cells were resuspended per 100 μL dye solution.

### Statistical analysis

Statistical analysis was performed using SPSS 21.0 (IBM Corp., Armonk, NY). The measurement data were described as mean ± standard deviation. Data between the two groups were compared using an unpaired *t*-test. Data among multiple groups were assessed by one-way analysis of variance (ANOVA) or two-way ANOVA, followed by Tukey’s post hoc tests with corrections for multiple comparisons. A value of *p* < 0.05 was statistically significant.

### Reporting summary

Further information on research design is available in the Nature Research Reporting Summary linked to this article.

### Supplementary information

Supplementary information

Reporting summary

## Data Availability

The data supporting this study’s findings are available on request from the corresponding author.
