# Supplementary material for: The antidepressive mechanism of Longya Lilium combined with Fluoxetine in mice with depression-like behaviors
Source: NPJ Syst Biol Appl. 2024 Jan 13;10:5. doi: 10.1038/s41540-024-00329-5 (PMC10787738; doi:10.1038/s41540-024-00329-5)
Supplement: Supplementary file 1 — Supplementary information [file 41540_2024_329_MOESM1_ESM.pdf]

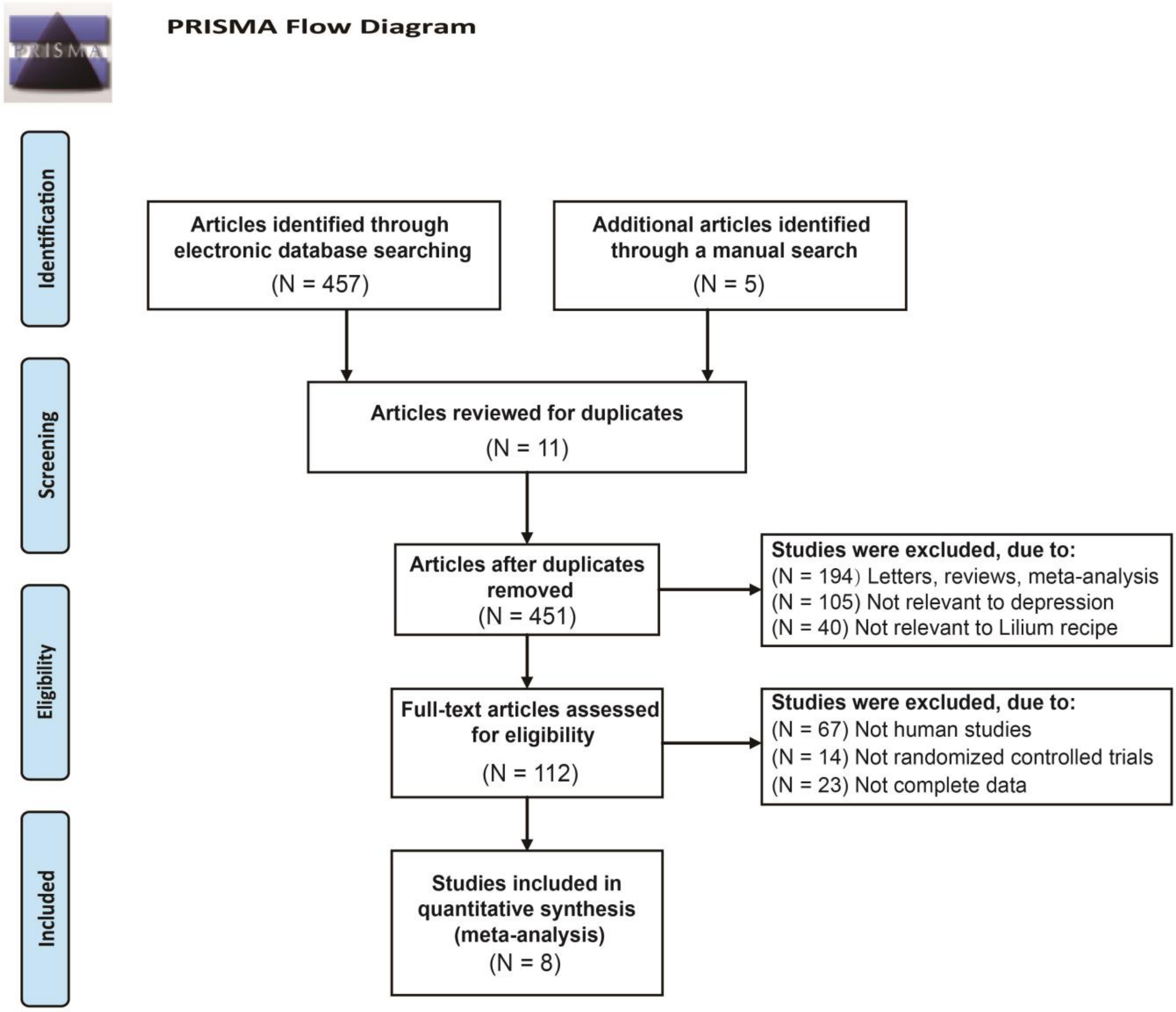

Supplementary Figure 1. Flow diagram of the literature screening process.

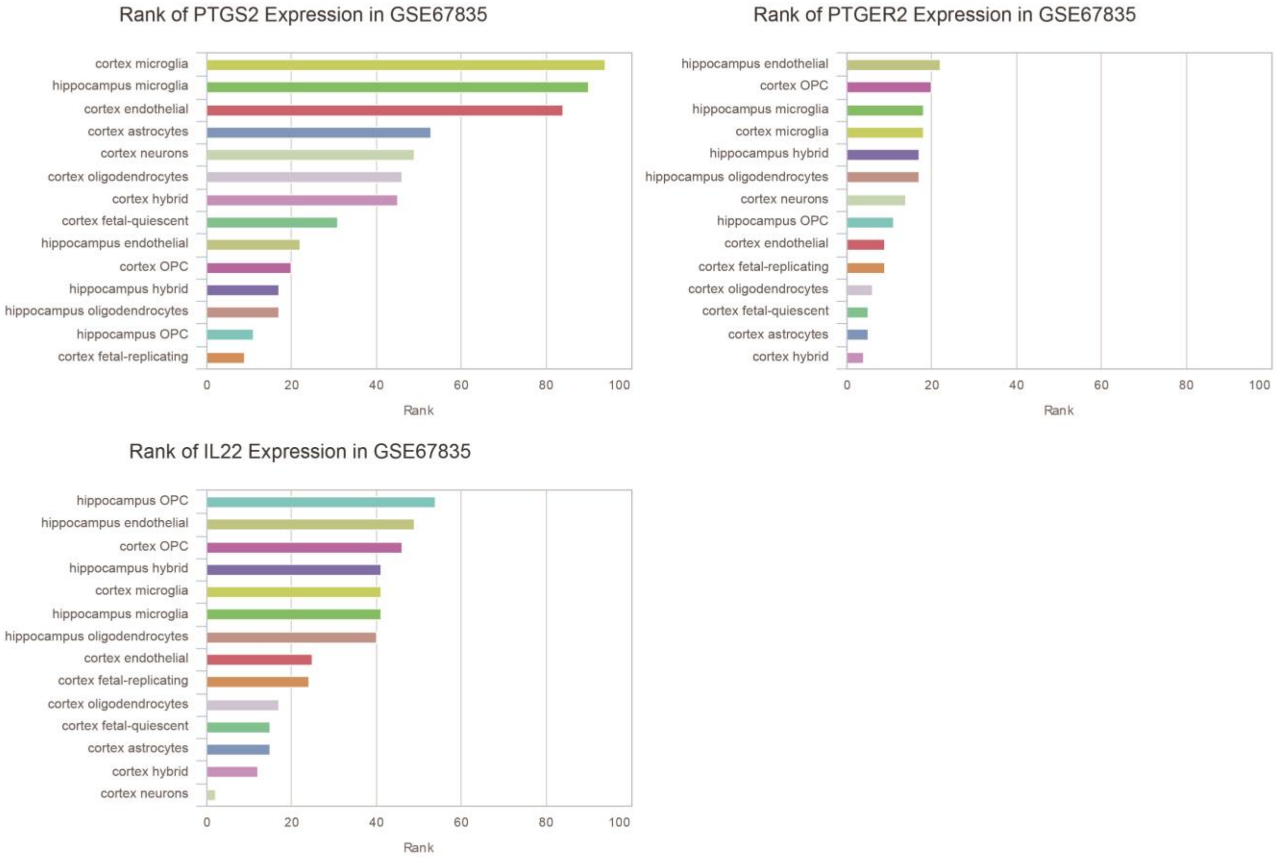

Supplementary Figure 2. Distribution of COX-2, PGE2, and IL-22 in brain tissue.

Supplementary Table 1 The baseline characteristics for the included studies

First author (Publication year)	Medication treatment		Treatment time	Sample size		Gender (M/F)		Age	
	control	Lilium saponins		control	Lilium saponins	control	Lilium saponins	control	Lilium saponins
Yang M M (2020)	Fluoxetine	Fluoxetine + Lilium saponins	8 weeks	35	35	20/15	21/14	53.75 ±8.25	53.18 ± 8.16
Huang H T (2020)	Fluoxetine	Fluoxetine + Lilium saponins	8 weeks	40	40	22/18	21/19	38.3 ± 12.6	37.8 ± 12.3
Jin Y M (2018)	Fluoxetine	Fluoxetine + Lilium saponins	8 weeks	36	36	9/27	8/28	43.75 ± 11.91	44.17 ± 12.07
Li E H (2016)	Fluoxetine	Fluoxetine + Lilium saponins	4 weeks	60	60	26/34	27/33	69.1 ± 6.2	69.2 ± 6.8
Zheng Z H (2014)	Fluoxetine	Fluoxetine + Lilium saponins	8 weeks	80	80	35/45	37/43	36.23 ±5.20	36.58 ± 5.26
Li L N (2014)	Fluoxetine	Fluoxetine + Lilium saponins	6 weeks	34	34	18/16	20/14	52.3 ± 4.5	51.2 ± 3.5
Han Y Q (2010)	Fluoxetine	Fluoxetine + Lilium saponins	8 weeks	60	60	58/62		52.4 ±8.5	

Ma Q M (2009)	Fluoxetine	Fluoxetine + Lilium saponins	6 weeks	20	26	24/22	45-76

Supplementary references

Yang, M.M., Zhang, H.L., 2020. [Effect of Baihe Dihuang decoction in the treatment of post-stroke depression]. Chin J Mod Drug Appl 14 (20), 3.

Huang, H.T., 2020. [Study on the effect of Ganmai Datao Decoction and Lily Anemone Decoction in treating depression]. Contemporary Medicine Forum 18 (13), 192-193.

Jin, Y.M., 2018. [Clinical observation of Baihe Zhimu decoction combined with Ganmai Dazao decoction and fluoxetine hydrochloride in the treatment of depression]. China's Naturopathy 26 (11), 73-74.

Li, E.H., 2016. [Baihe Ningshen Tang Combined Low-dose Fluoxetine Treatment Depression after Stroke Randomized Controlled Study]. Journal of Practical Traditional Chinese Internal Medicine 30 (7), 55-57.

Zheng, Z.H., 2014. [Randomized Parallel Controlled Study of Jieyu Decoction Combined with Western Medicine in Differentiation Treatment of Depression Syndrome]. J Journal of Practical Traditional Chinese Internal Medicine 28 (7), 99-101.

Li, L.N., Gao, L.Y., 2014. [34 Cases of Depression Treated with Modified Baihe Dihuang Decoction]. Henan Traditional Chinese Medicine 34 (5), 803-804.

Han, Y.Q., Liu, D.Y., Ji, S.M., Mi, J.L., 2010. [Clinical observation of Baihe Ningshen decoction combined with low-dose fluoxetine in the treatment of 60 cases of post-stroke depression]. Shandong Medical Journal 50 (23), 69-70.

Ma, Q.M., Yao, W.Y., Wen, T.X., Zhu, C.X., 2009. [Combine Traditional Chinese and Western Medicine Treatment of Depression after Cerebral Infarction]. Journal of Practical Traditional Chinese Internal Medicine 23 (12), 94-95.

Supplementary Table 2 Correlation of 6 key target genes with drugs and disease

Genes	Lilium saponins component	Interaction count	Inference score
COX-2	MOL000358, MOL001947	3	199.57
SLC6A4	MOL000358	27	182.31
KCNH2	MOL000358	10	169.05
CASP3	MOL000358	8	340.62
NR3C2	MOL009465, MOL009473	7	108.05
BCL2	MOL000358	4	245.02

Note: Interaction count and inference score represent the values in the CTD database based on literature. The higher value indicates the stronger evidence.

Supplementary Table 3 Primer sequences for reverse transcription quantitative polymerase chain reaction

Gene	Sequence
COX-2	Forward: 5'-TCCCTGAAGCCGTACACATCA-3'<br>Reverse: 5'-TGGACGAGGTTTTTCCACCA'
PGE2	Forward: 5'-TGGAGGTGAATCCCGTGAGA-3'<br>Reverse: 5'-AAACTCGGTCACCTCCTTGC-3'
IL-22	Forward: 5'-TCCAACTTCCAGCAGCCATACATC-3'<br>Reverse: 5'-GCACTGATCCTTAGCACTGACTCC-3'
GAPDH	Forward: 5'-AGGTCGGTGTGAACGGATTTG -3'<br>Reverse: 5'-TGTAGACCATGTAGTTGAGGTCA-3'

Note: COX-2, cyclooxygenase-2; PGE2, prostaglandin E2; IL-22, interleukin-22; GAPDH, glyceraldehyde-3-phosphate dehydrogenase

WB images

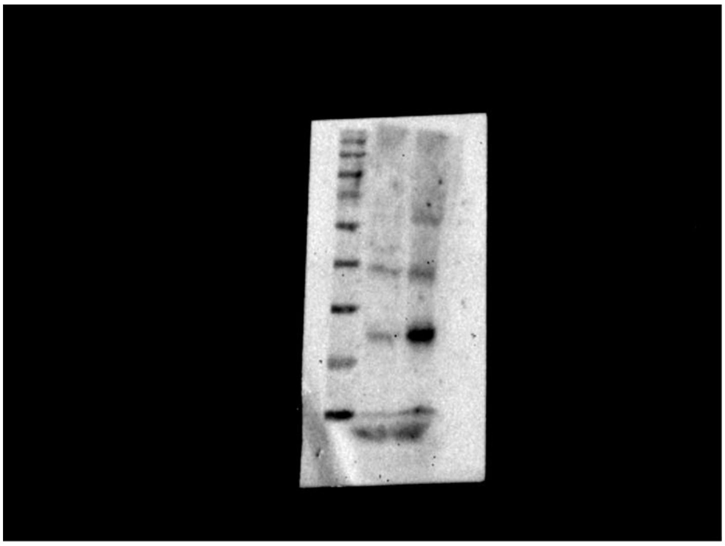

Supplementary Figure 3(Figure4E-1-1)

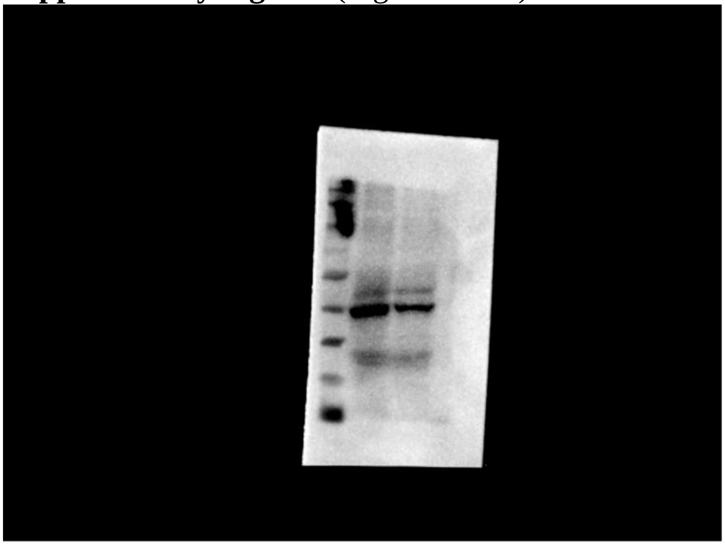

Supplementary Figure 4(Figure4E-1-2)

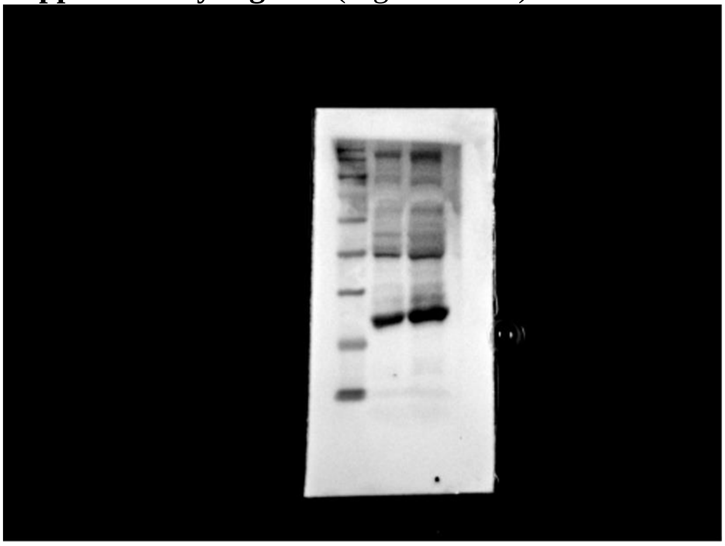

Supplementary Figure 5(Figure4E-1-3)

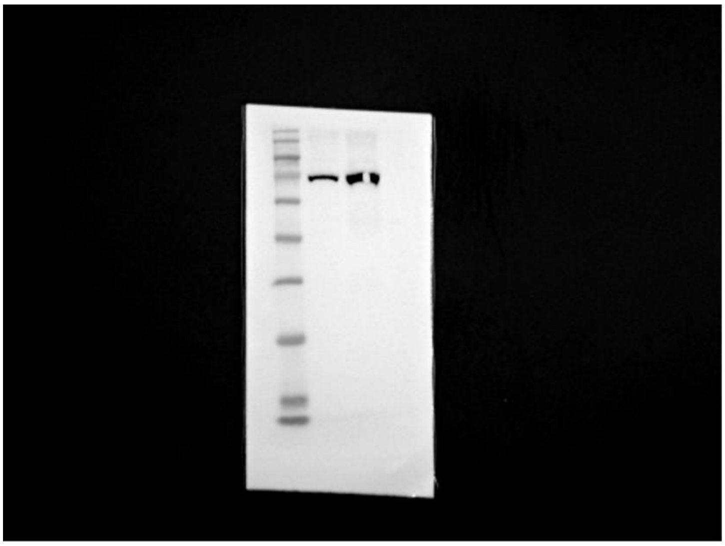

Supplementary Figure 6(Figure4E-2-1)

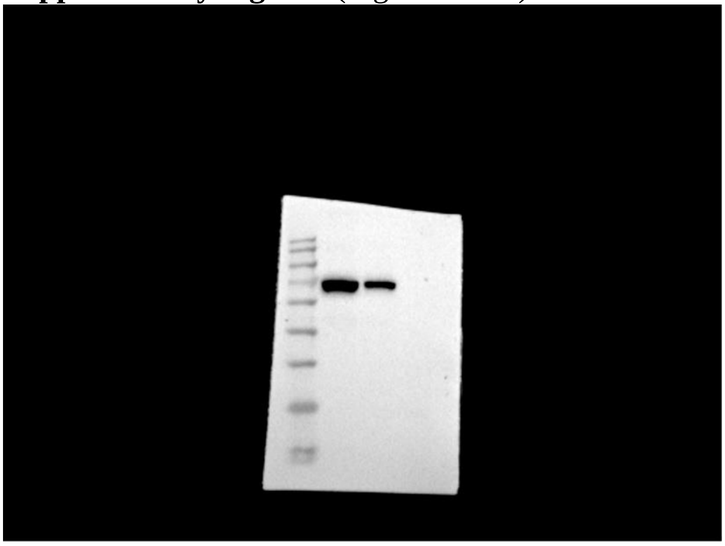

Supplementary Figure 7(Figure4E-2-2)

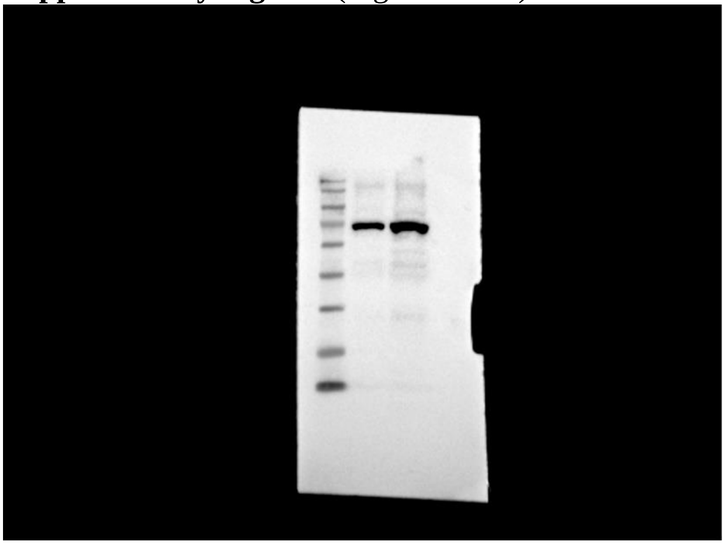

Supplementary Figure 8(Figure4E-2-3)

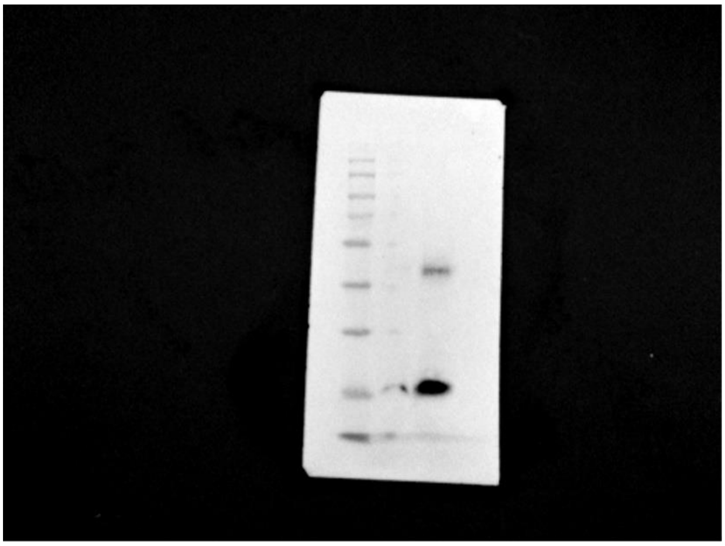

Supplementary Figure 9(Figure4E-3-1)

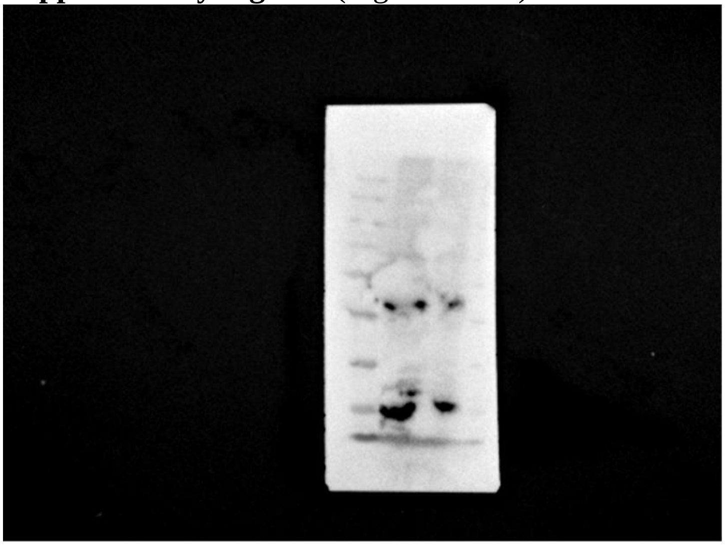

Supplementary Figure 10(Figure4E-3-2)

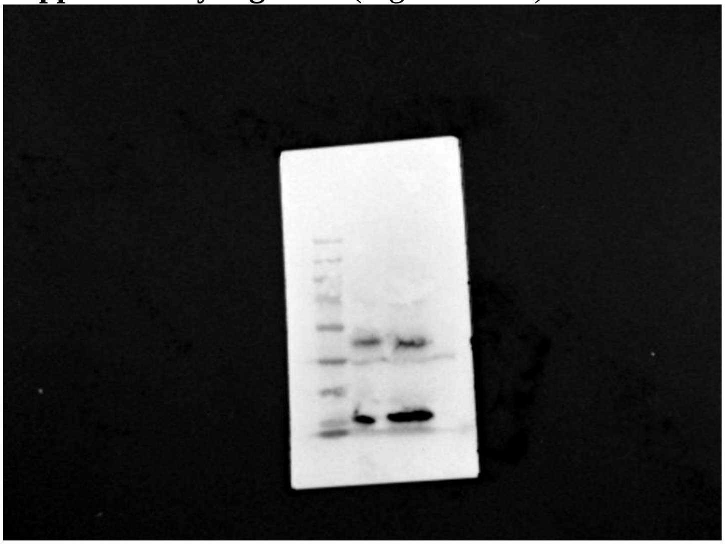

Supplementary Figure 11(Figure4E-3-3)

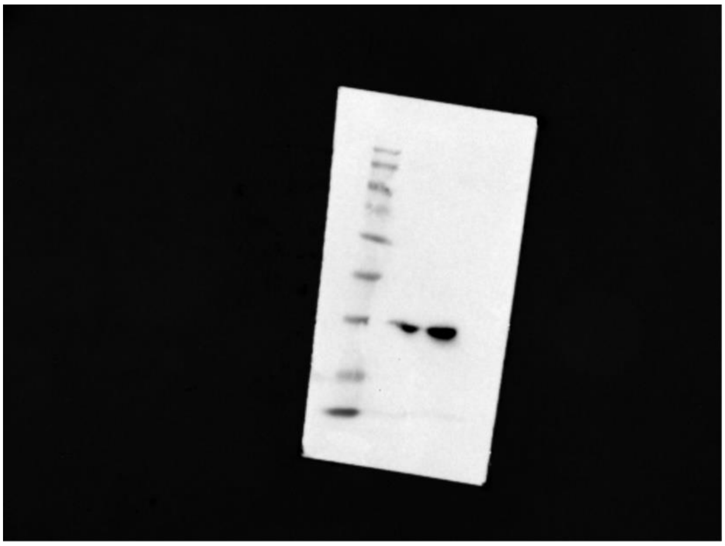
Supplementary Figure 12(Figure4E-4-1)
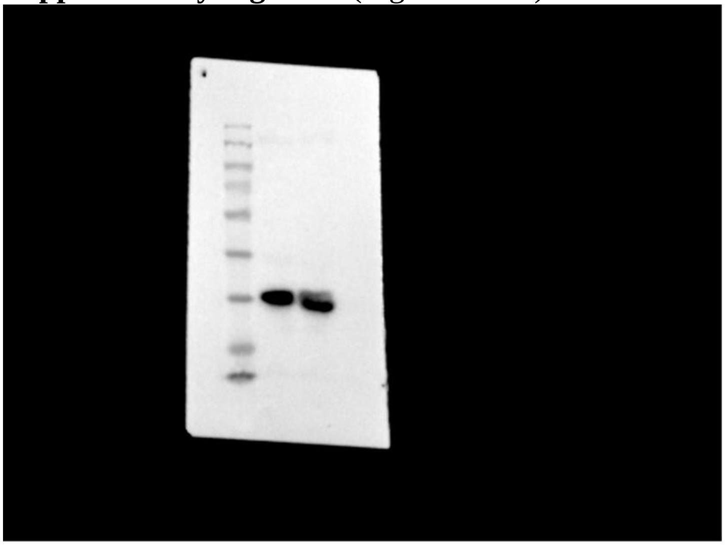
Supplementary Figure 13(Figure4E-4-2)
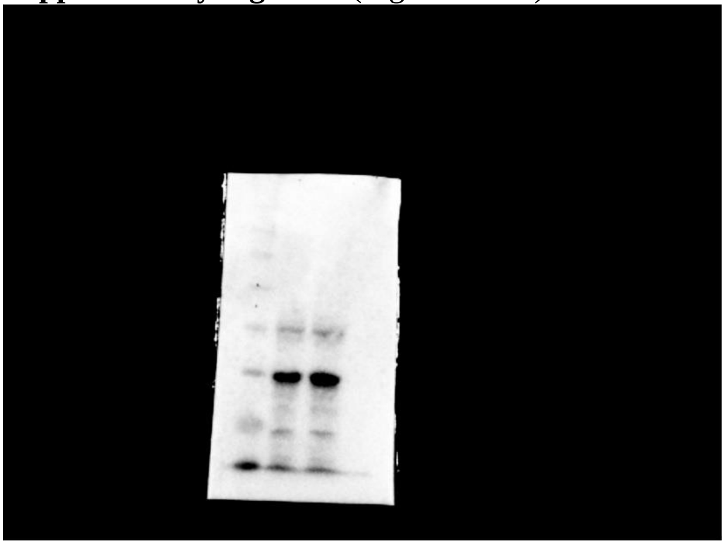
Supplementary Figure 14(Figure4E-4-3)

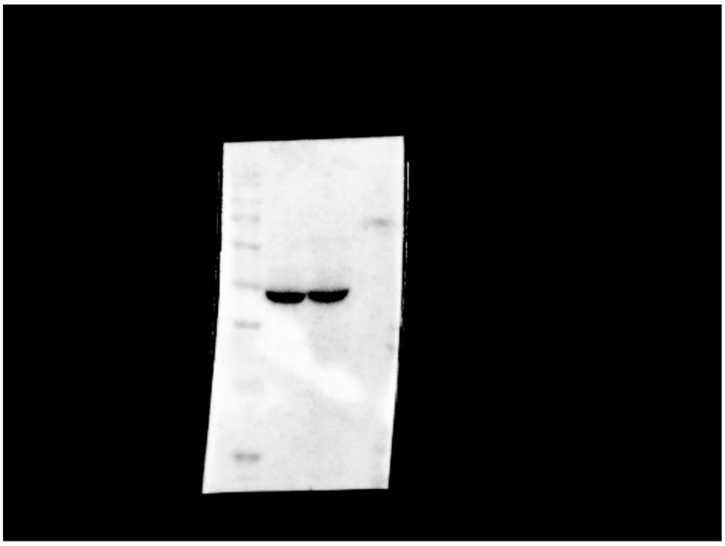

Supplementary Figure 15(Figure4E-5-1)

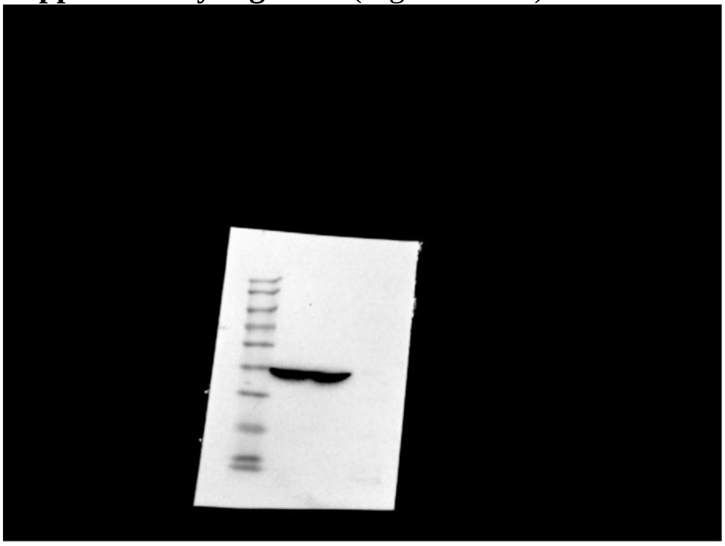

Supplementary Figure 16(Figure4E-5-2)

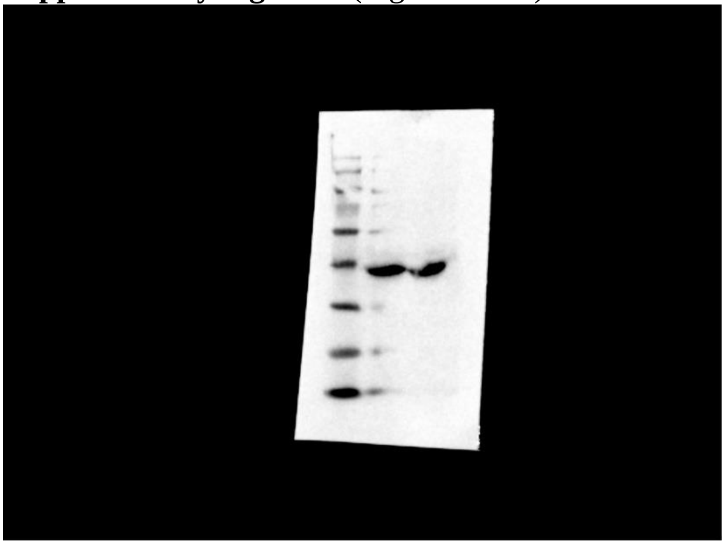

Supplementary Figure 17(Figure4E-5-3)

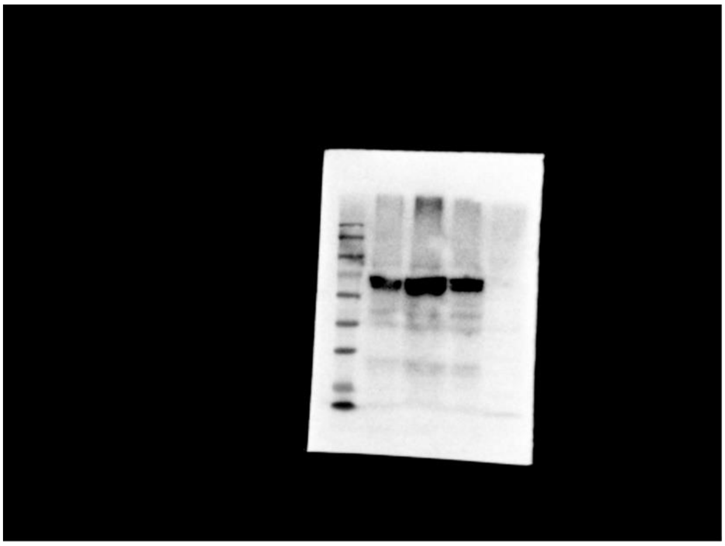

Supplementary Figure 18(Figure5A-1)

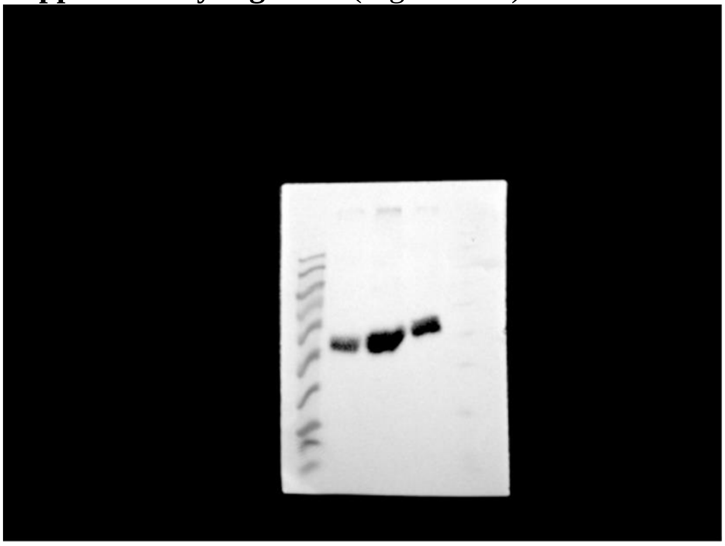

Supplementary Figure 19(Figure5A-2)

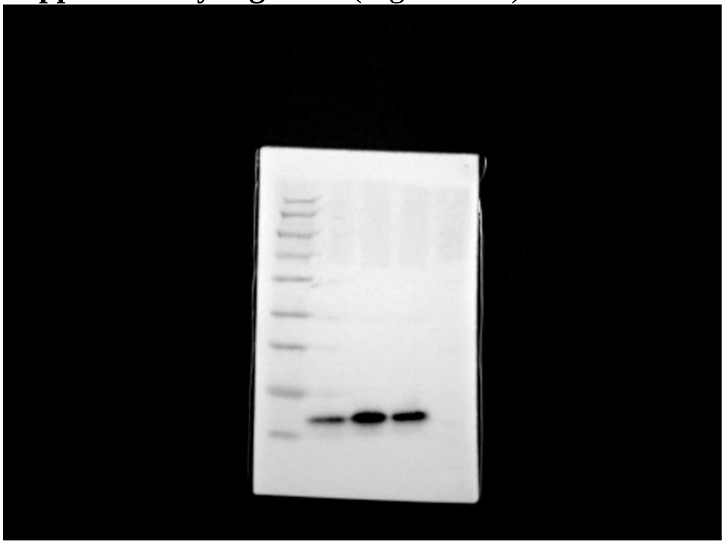

Supplementary Figure 20(Figure5A-3)

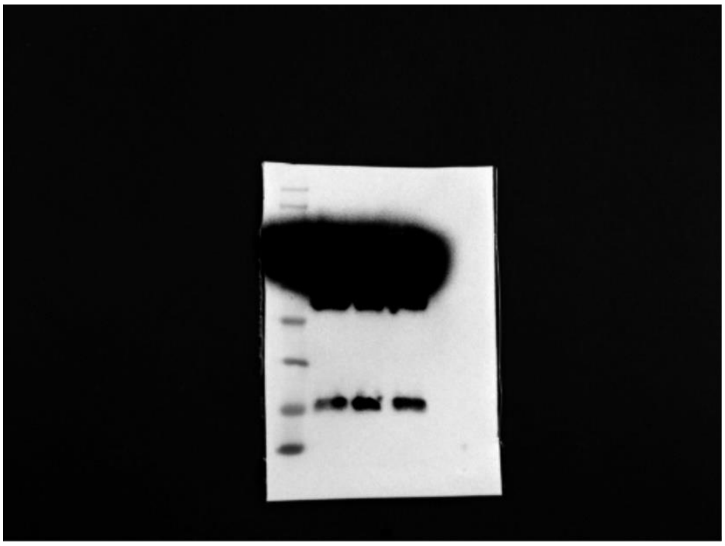

Supplementary Figure 21(Figure5A-4)

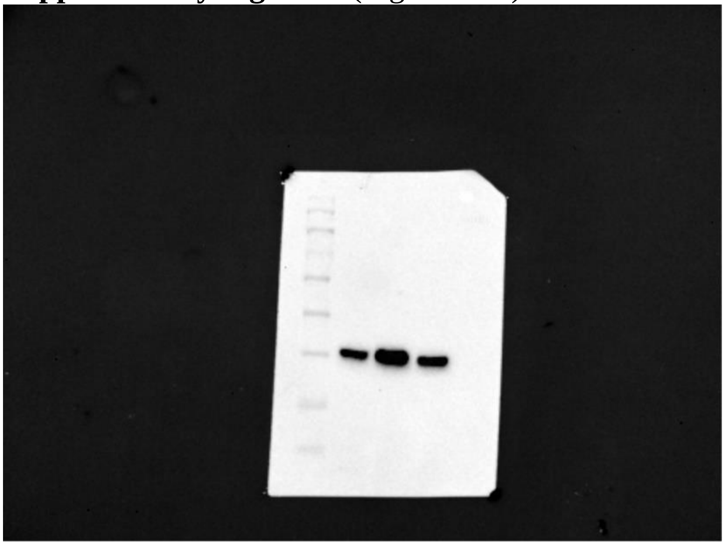

Supplementary Figure 22(Figure5A-5)

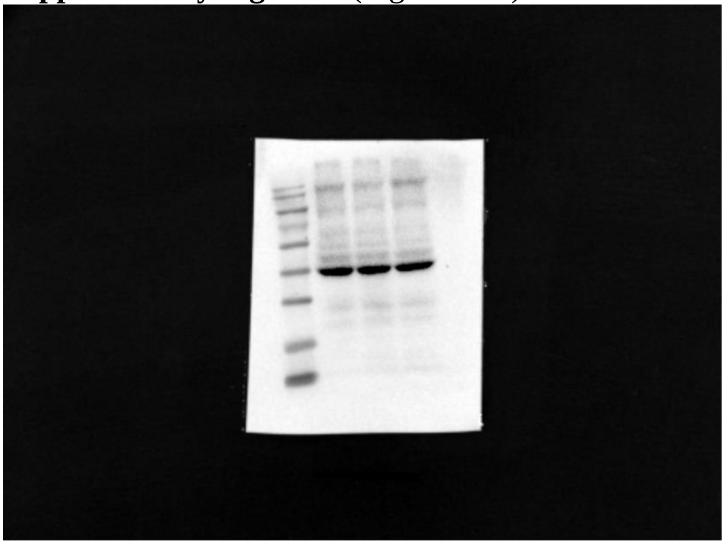

Supplementary Figure 23(Figure5A-6)

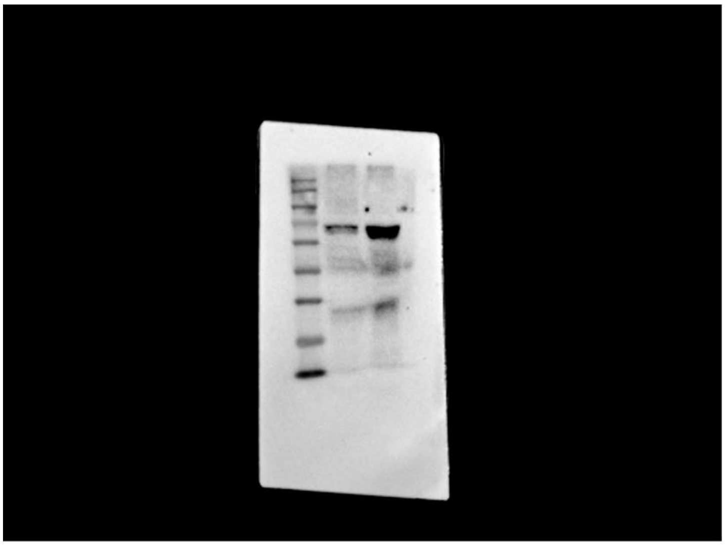

Supplementary Figure 24(Figure6E-1-1)

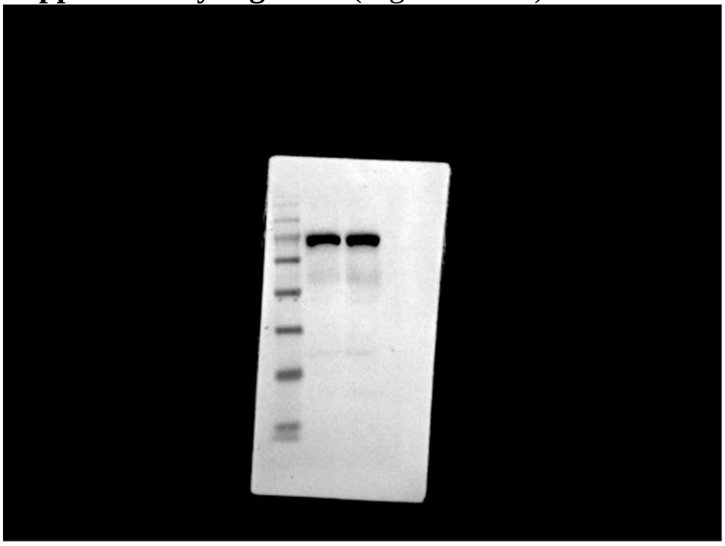

Supplementary Figure 25(Figure6E-1-2)

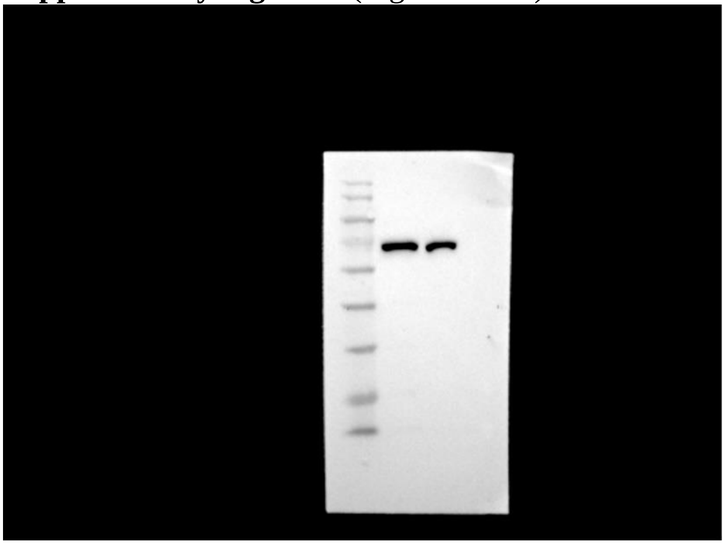

Supplementary Figure 26(Figure6E-1-3)

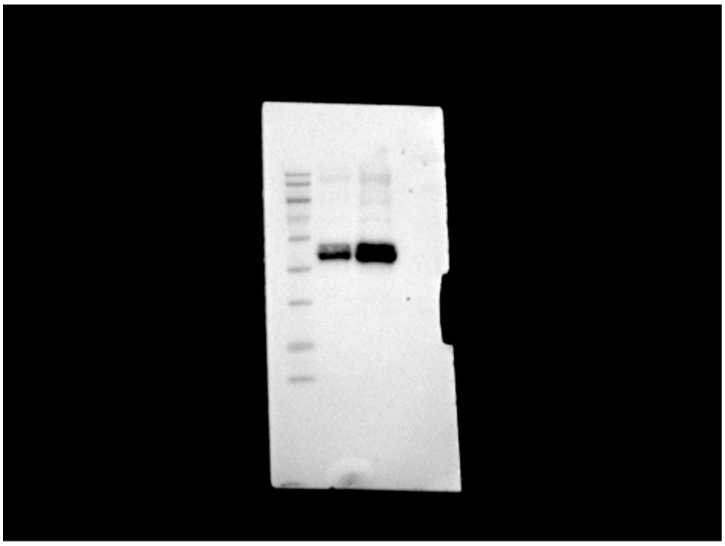

Supplementary Figure 27(Figure6E-2-1)

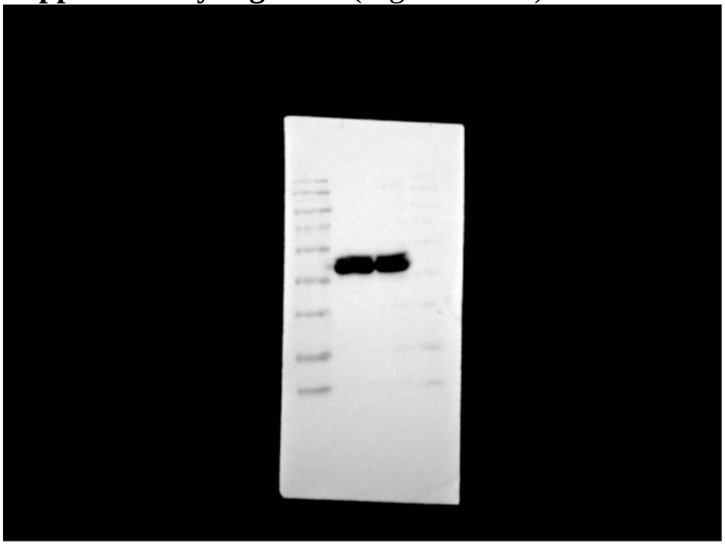

Supplementary Figure 28(Figure6E-2-2)

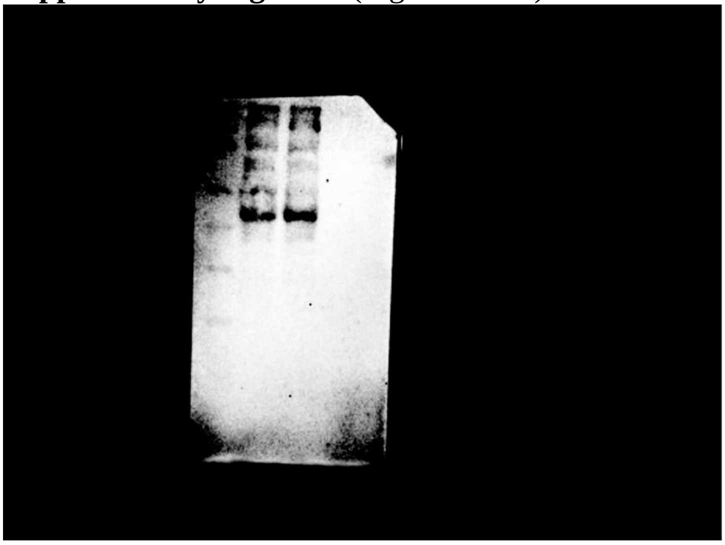

Supplementary Figure 29(Figure6E-2-3)

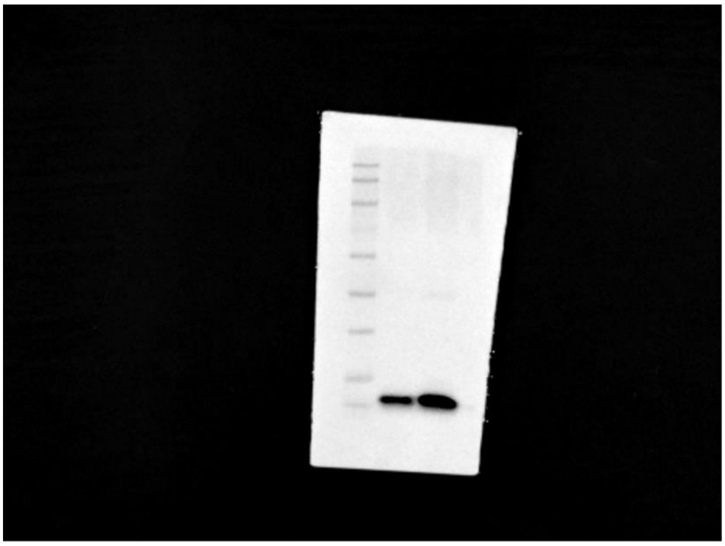

Supplementary Figure 30(Figure6E-3-1)

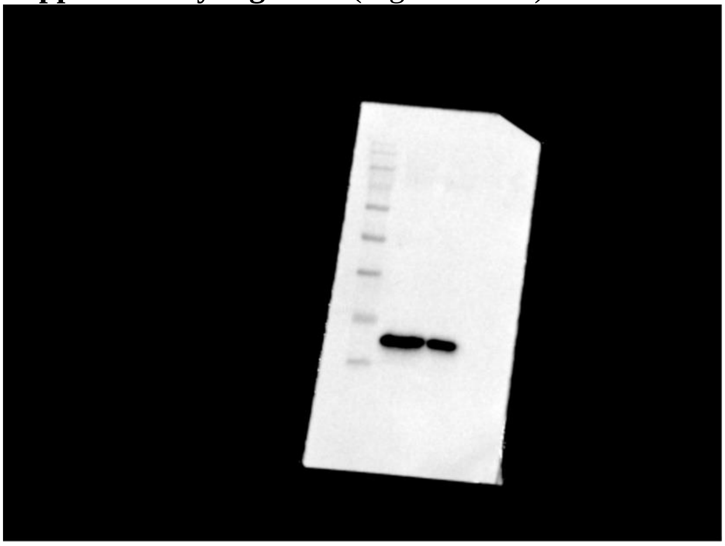

Supplementary Figure 31(Figure6E-3-2)

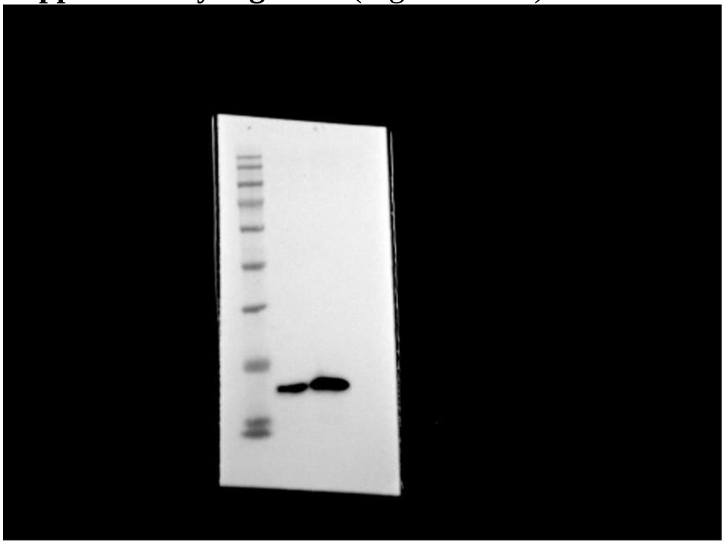

Supplementary Figure 32(Figure6E-3-3)

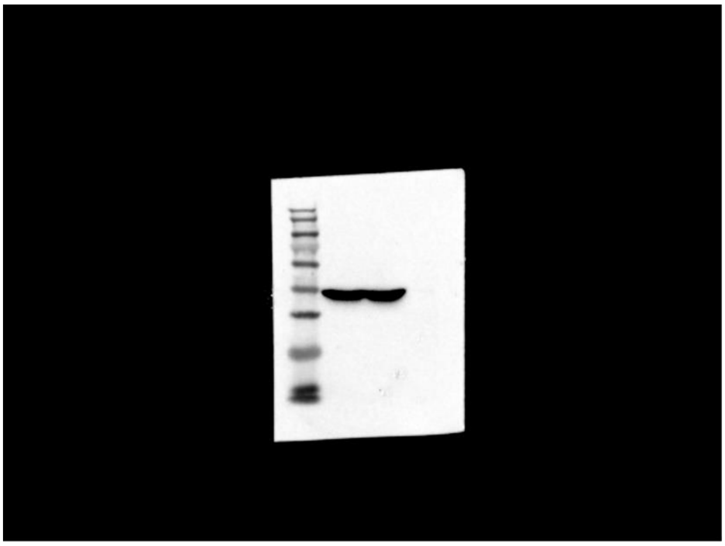

Supplementary Figure 33(Figure6E-4-1)

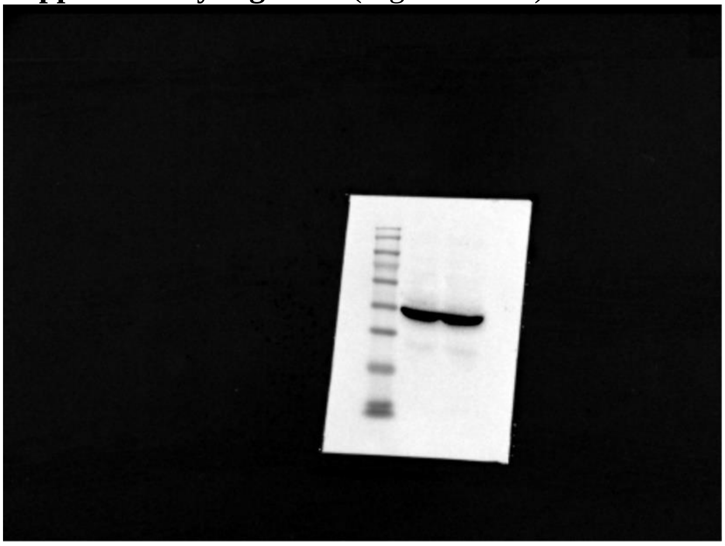

Supplementary Figure 34(Figure6E-4-2)

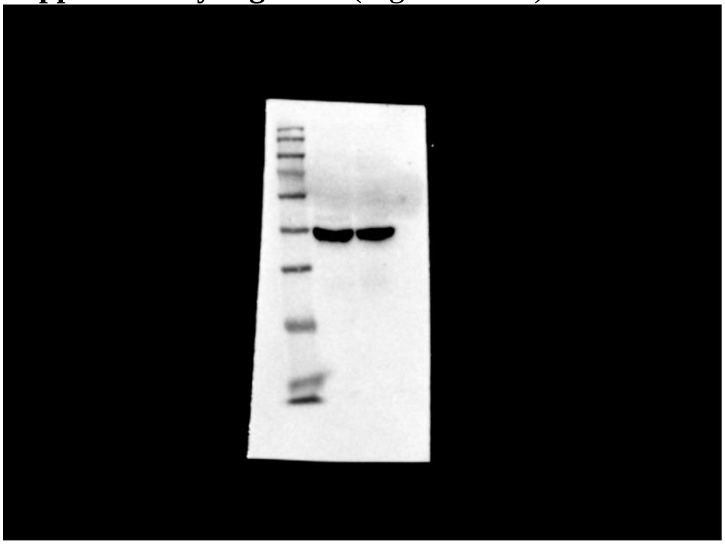

Supplementary Figure 35(Figure6E-4-3)
