# Supplementary material for: The antidepressive mechanism of Longya Lilium combined with Fluoxetine in mice with depression-like behaviors
Source: NPJ Syst Biol Appl. 2024 Jan 13;10:5. doi: 10.1038/s41540-024-00329-5 (PMC10787738; doi:10.1038/s41540-024-00329-5)
Supplement: Supplementary file 2 — Reporting summary [file 41540_2024_329_MOESM2_ESM.pdf]

# nature portfolio

Corresponding author(s): Yanwu Ling

Last updated by author(s): Oct 28, 2023

# Reporting Summary

Nature Portfolio wishes to improve the reproducibility of the work that we publish. This form provides structure for consistency and transparency in reporting. For further information on Nature Portfolio policies, see our Editorial Policies and the Editorial Policy Checklist.

## Statistics

For all statistical analyses, confirm that the following items are present in the figure legend, table legend, main text, or Methods section.

| n/a | Confirmed | |
|---|---|---|
| ☐ | ☒ | The exact sample size (*n*) for each experimental group/condition, given as a discrete number and unit of measurement |
| ☐ | ☒ | A statement on whether measurements were taken from distinct samples or whether the same sample was measured repeatedly |
| ☐ | ☒ | The statistical test(s) used AND whether they are one- or two-sided<br>*Only common tests should be described solely by name; describe more complex techniques in the Methods section.* |
| ☒ | ☐ | A description of all covariates tested |
| ☒ | ☐ | A description of any assumptions or corrections, such as tests of normality and adjustment for multiple comparisons |
| ☐ | ☒ | A full description of the statistical parameters including central tendency (e.g. means) or other basic estimates (e.g. regression coefficient) AND variation (e.g. standard deviation) or associated estimates of uncertainty (e.g. confidence intervals) |
| ☐ | ☒ | For null hypothesis testing, the test statistic (e.g. $F$, $t$, $r$) with confidence intervals, effect sizes, degrees of freedom and $P$ value noted<br>*Give P values as exact values whenever suitable.* |
| ☒ | ☐ | For Bayesian analysis, information on the choice of priors and Markov chain Monte Carlo settings |
| ☒ | ☐ | For hierarchical and complex designs, identification of the appropriate level for tests and full reporting of outcomes |
| ☒ | ☐ | Estimates of effect sizes (e.g. Cohen's $d$, Pearson's $r$), indicating how they were calculated |

*Our web collection on statistics for biologists contains articles on many of the points above.*

## Software and code

Policy information about availability of computer code

| | |
|---|---|
| Data collection | The data supporting this study's findings are available on request from the corresponding author. |
| Data analysis | The data supporting this study's findings are available on request from the corresponding author. |

For manuscripts utilizing custom algorithms or software that are central to the research but not yet described in published literature, software must be made available to editors and reviewers. We strongly encourage code deposition in a community repository (e.g. GitHub). See the Nature Portfolio guidelines for submitting code & software for further information.

## Data

Policy information about availability of data

All manuscripts must include a data availability statement. This statement should provide the following information, where applicable:

- Accession codes, unique identifiers, or web links for publicly available datasets
- A description of any restrictions on data availability
- For clinical datasets or third party data, please ensure that the statement adheres to our policy

The data supporting this study's findings are available on request from the corresponding author.

## Research involving human participants, their data, or biological material

Policy information about studies with human participants or human data. See also policy information about sex, gender (identity/presentation), and sexual orientation and race, ethnicity and racism.

| | |
|---|---|
| Reporting on sex and gender | 120 SPF C57BL/6J male mice (aged 9-12 weeks old, weighing 18-21 g) were used to develop a model of depression based on the CUMS method. The stress paradigm consisted of the following stressors: swimming in ice water (5 min), food and water deprivation (24 h for each), tail pinch (1 min), shaking (once/s, 15 min), reversal of day and night and bondage (5 min each time) for a total of 24 days. One of these stressors was randomly arranged every day, and each stimulus was performed 21 times so that the mice could not predict the occurrence of the stimulus. Ten mice were used as normal controls, and 110 were stimulated to establish a depression model. After 5 weeks, the behavioral test was performed, and the serum sample was collected for biochemical testing. |
| Reporting on race, ethnicity, or other socially relevant groupings | 120 SPF C57BL/6J male mice (aged 9-12 weeks old, weighing 18-21 g) were used to develop a model of depression based on the CUMS method. The stress paradigm consisted of the following stressors: swimming in ice water (5 min), food and water deprivation (24 h for each), tail pinch (1 min), shaking (once/s, 15 min), reversal of day and night and bondage (5 min each time) for a total of 24 days. One of these stressors was randomly arranged every day, and each stimulus was performed 21 times so that the mice could not predict the occurrence of the stimulus. Ten mice were used as normal controls, and 110 were stimulated to establish a depression model. After 5 weeks, the behavioral test was performed, and the serum sample was collected for biochemical testing. |
| Population characteristics | 120 SPF C57BL/6J male mice (aged 9-12 weeks old, weighing 18-21 g) were used to develop a model of depression based on the CUMS method. The stress paradigm consisted of the following stressors: swimming in ice water (5 min), food and water deprivation (24 h for each), tail pinch (1 min), shaking (once/s, 15 min), reversal of day and night and bondage (5 min each time) for a total of 24 days. One of these stressors was randomly arranged every day, and each stimulus was performed 21 times so that the mice could not predict the occurrence of the stimulus. Ten mice were used as normal controls, and 110 were stimulated to establish a depression model. After 5 weeks, the behavioral test was performed, and the serum sample was collected for biochemical testing. |
| Recruitment | 120 SPF C57BL/6J male mice (aged 9-12 weeks old, weighing 18-21 g) were used to develop a model of depression based on the CUMS method. The stress paradigm consisted of the following stressors: swimming in ice water (5 min), food and water deprivation (24 h for each), tail pinch (1 min), shaking (once/s, 15 min), reversal of day and night and bondage (5 min each time) for a total of 24 days. One of these stressors was randomly arranged every day, and each stimulus was performed 21 times so that the mice could not predict the occurrence of the stimulus. Ten mice were used as normal controls, and 110 were stimulated to establish a depression model. After 5 weeks, the behavioral test was performed, and the serum sample was collected for biochemical testing. |
| Ethics oversight | The current study was performed with the approval of the Ethics Committee of Youjiang Medical University for Nationalities and performed strictly with the Guide for the Care and Use of Laboratory Animals published by the US National Institutes of Health. |

Note that full information on the approval of the study protocol must also be provided in the manuscript.

# Field-specific reporting

Please select the one below that is the best fit for your research. If you are not sure, read the appropriate sections before making your selection.

☒ Life sciences ☐ Behavioural & social sciences ☐ Ecological, evolutionary & environmental sciences

For a reference copy of the document with all sections, see nature.com/documents/nr-reporting-summary-flat.pdf

# Life sciences study design

All studies must disclose on these points even when the disclosure is negative.

| | |
|---|---|
| Sample size | 120 SPF C57BL/6J male mice (aged 9-12 weeks old, weighing 18-21 g) were used to develop a model of depression based on the CUMS method. The stress paradigm consisted of the following stressors: swimming in ice water (5 min), food and water deprivation (24 h for each), tail pinch (1 min), shaking (once/s, 15 min), reversal of day and night and bondage (5 min each time) for a total of 24 days. One of these stressors was randomly arranged every day, and each stimulus was performed 21 times so that the mice could not predict the occurrence of the stimulus. Ten mice were used as normal controls, and 110 were stimulated to establish a depression model. After 5 weeks, the behavioral test was performed, and the serum sample was collected for biochemical testing. |
| Data exclusions | 120 SPF C57BL/6J male mice (aged 9-12 weeks old, weighing 18-21 g) were used to develop a model of depression based on the CUMS method. The stress paradigm consisted of the following stressors: swimming in ice water (5 min), food and water deprivation (24 h for each), tail pinch (1 min), shaking (once/s, 15 min), reversal of day and night and bondage (5 min each time) for a total of 24 days. One of these stressors was randomly arranged every day, and each stimulus was performed 21 times so that the mice could not predict the occurrence of the stimulus. Ten mice were used as normal controls, and 110 were stimulated to establish a depression model. After 5 weeks, the behavioral test was performed, and the serum sample was collected for biochemical testing. |
| Replication | 120 SPF C57BL/6J male mice (aged 9-12 weeks old, weighing 18-21 g) were used to develop a model of depression based on the CUMS method. The stress paradigm consisted of the following stressors: swimming in ice water (5 min), food and water deprivation (24 h for each), tail pinch (1 min), shaking (once/s, 15 min), reversal of day and night and bondage (5 min each time) for a total of 24 days. One of these |

| | |
|---|---|
| | stressors was randomly arranged every day, and each stimulus was performed 21 times so that the mice could not predict the occurrence of the stimulus. Ten mice were used as normal controls, and 110 were stimulated to establish a depression model. After 5 weeks, the behavioral test was performed, and the serum sample was collected for biochemical testing. |
| Randomization | 120 SPF C57BL/6J male mice (aged 9-12 weeks old, weighing 18-21 g) were used to develop a model of depression based on the CUMS method. The stress paradigm consisted of the following stressors: swimming in ice water (5 min), food and water deprivation (24 h for each), tail pinch (1 min), shaking (once/s, 15 min), reversal of day and night and bondage (5 min each time) for a total of 24 days. One of these stressors was randomly arranged every day, and each stimulus was performed 21 times so that the mice could not predict the occurrence of the stimulus. Ten mice were used as normal controls, and 110 were stimulated to establish a depression model. After 5 weeks, the behavioral test was performed, and the serum sample was collected for biochemical testing. |
| Blinding | 120 SPF C57BL/6J male mice (aged 9-12 weeks old, weighing 18-21 g) were used to develop a model of depression based on the CUMS method. The stress paradigm consisted of the following stressors: swimming in ice water (5 min), food and water deprivation (24 h for each), tail pinch (1 min), shaking (once/s, 15 min), reversal of day and night and bondage (5 min each time) for a total of 24 days. One of these stressors was randomly arranged every day, and each stimulus was performed 21 times so that the mice could not predict the occurrence of the stimulus. Ten mice were used as normal controls, and 110 were stimulated to establish a depression model. After 5 weeks, the behavioral test was performed, and the serum sample was collected for biochemical testing. |

# Reporting for specific materials, systems and methods

We require information from authors about some types of materials, experimental systems and methods used in many studies. Here, indicate whether each material, system or method listed is relevant to your study. If you are not sure if a list item applies to your research, read the appropriate section before selecting a response.

## Materials & experimental systems

| n/a | Involved in the study |
|---|---|
| ☐ | ☒ Antibodies |
| ☒ | ☐ Eukaryotic cell lines |
| ☒ | ☐ Palaeontology and archaeology |
| ☐ | ☒ Animals and other organisms |
| ☒ | ☐ Clinical data |
| ☒ | ☐ Dual use research of concern |
| ☒ | ☐ Plants |

## Methods

| n/a | Involved in the study |
|---|---|
| ☒ | ☐ ChIP-seq |
| ☐ | ☒ Flow cytometry |
| ☒ | ☐ MRI-based neuroimaging |

## Antibodies

| | |
|---|---|
| Antibodies used | The mouse hippocampal tissue sections were fixed with 4% paraformaldehyde, permeabilized in 0.3% Triton X-100, and blocked with 1% bovine serum albumin. After that, the sections were probed with primary antibody Iba1 (A12391, 1:100, Abclonal Technology, Inc.) overnight at 4°C. Following PBS washing, the sections were re-probed with corresponding fluorescent secondary antibodies against AS011 and AS007 (1:100, Abclonal). Finally, the sections were observed under a confocal laser scanning microscope (FluoView FV10i, Olympus Optical Co., Ltd, Tokyo, Japan). Five sections were randomly selected from each mouse, and three visual fields were randomly selected from each section for a photograph. ImageJ software (National Institutes of Health, Bethesda, Maryland) was used for fluorescence intensity analysis. |
| Validation | The mouse hippocampal tissue sections were fixed with 4% paraformaldehyde, permeabilized in 0.3% Triton X-100, and blocked with 1% bovine serum albumin. After that, the sections were probed with primary antibody Iba1 (A12391, 1:100, Abclonal Technology, Inc.) overnight at 4°C. Following PBS washing, the sections were re-probed with corresponding fluorescent secondary antibodies against AS011 and AS007 (1:100, Abclonal). Finally, the sections were observed under a confocal laser scanning microscope (FluoView FV10i, Olympus Optical Co., Ltd, Tokyo, Japan). Five sections were randomly selected from each mouse, and three visual fields were randomly selected from each section for a photograph. ImageJ software (National Institutes of Health, Bethesda, Maryland) was used for fluorescence intensity analysis. |

## Animals and other research organisms

Policy information about studies involving animals; ARRIVE guidelines recommended for reporting animal research, and Sex and Gender in Research

| | |
|---|---|
| Laboratory animals | 120 SPF C57BL/6J male mice (aged 9-12 weeks old, weighing 18-21 g) were used to develop a model of depression based on the CUMS method. The stress paradigm consisted of the following stressors: swimming in ice water (5 min), food and water deprivation (24 h for each), tail pinch (1 min), shaking (once/s, 15 min), reversal of day and night and bondage (5 min each time) for a total of 24 days. One of these stressors was randomly arranged every day, and each stimulus was performed 21 times so that the mice could not predict the occurrence of the stimulus. Ten mice were used as normal controls, and 110 were stimulated to establish a depression model. After 5 weeks, the behavioral test was performed, and the serum sample was collected for biochemical testing. |
| Wild animals | N/A |
| Reporting on sex | 120 SPF C57BL/6J male mice (aged 9-12 weeks old, weighing 18-21 g) were used to develop a model of depression based on the |

| | |
|---|---|
| Reporting on sex | CUMS method. The stress paradigm consisted of the following stressors: swimming in ice water (5 min), food and water deprivation (24 h for each), tail pinch (1 min), shaking (once/s, 15 min), reversal of day and night and bondage (5 min each time) for a total of 24 days. One of these stressors was randomly arranged every day, and each stimulus was performed 21 times so that the mice could not predict the occurrence of the stimulus. Ten mice were used as normal controls, and 110 were stimulated to establish a depression model. After 5 weeks, the behavioral test was performed, and the serum sample was collected for biochemical testing. |
| Field-collected samples | 120 SPF C57BL/6J male mice (aged 9-12 weeks old, weighing 18-21 g) were used to develop a model of depression based on the CUMS method. The stress paradigm consisted of the following stressors: swimming in ice water (5 min), food and water deprivation (24 h for each), tail pinch (1 min), shaking (once/s, 15 min), reversal of day and night and bondage (5 min each time) for a total of 24 days. One of these stressors was randomly arranged every day, and each stimulus was performed 21 times so that the mice could not predict the occurrence of the stimulus. Ten mice were used as normal controls, and 110 were stimulated to establish a depression model. After 5 weeks, the behavioral test was performed, and the serum sample was collected for biochemical testing. |
| Ethics oversight | The current study was performed with the approval of the Ethics Committee of Youjiang Medical University for Nationalities and performed strictly with the Guide for the Care and Use of Laboratory Animals published by the US National Institutes of Health. |

Note that full information on the approval of the study protocol must also be provided in the manuscript.

## Plants

| | |
|---|---|
| Seed stocks | N/A |
| Novel plant genotypes | N/A |
| Authentication | N/A |

## Flow Cytometry

### Plots

Confirm that:

☒ The axis labels state the marker and fluorochrome used (e.g. CD4-FITC).

☒ The axis scales are clearly visible. Include numbers along axes only for bottom left plot of group (a 'group' is an analysis of identical markers).

☒ All plots are contour plots with outliers or pseudocolor plots.

☒ A numerical value for number of cells or percentage (with statistics) is provided.

### Methodology

| | |
|---|---|
| Sample preparation | A flow cytometer assessed the cell apoptosis after 48 h of transfection. Following the instructions of Annexin-V-FITC Cell Apoptosis Detection Kit (CA1020, Beijing Solarbio Science & Technology Co., Ltd., Beijing, China), Annexin-V-FITC, PI, and HEPES buffer were prepared into Annexin-V-FITC/PI dye solution at a ratio of 1:2:50. A total of 1 × 106 cells were resuspended per 100 μL dye solution. |
| Instrument | A flow cytometer assessed the cell apoptosis after 48 h of transfection. Following the instructions of Annexin-V-FITC Cell Apoptosis Detection Kit (CA1020, Beijing Solarbio Science & Technology Co., Ltd., Beijing, China), Annexin-V-FITC, PI, and HEPES buffer were prepared into Annexin-V-FITC/PI dye solution at a ratio of 1:2:50. A total of 1 × 106 cells were resuspended per 100 μL dye solution. |
| Software | A flow cytometer assessed the cell apoptosis after 48 h of transfection. Following the instructions of Annexin-V-FITC Cell Apoptosis Detection Kit (CA1020, Beijing Solarbio Science & Technology Co., Ltd., Beijing, China), Annexin-V-FITC, PI, and HEPES buffer were prepared into Annexin-V-FITC/PI dye solution at a ratio of 1:2:50. A total of 1 × 106 cells were resuspended per 100 μL dye solution. |
| Cell population abundance | A flow cytometer assessed the cell apoptosis after 48 h of transfection. Following the instructions of Annexin-V-FITC Cell Apoptosis Detection Kit (CA1020, Beijing Solarbio Science & Technology Co., Ltd., Beijing, China), Annexin-V-FITC, PI, and HEPES buffer were prepared into Annexin-V-FITC/PI dye solution at a ratio of 1:2:50. A total of 1 × 106 cells were resuspended per 100 μL dye solution. |

| | |
|---|---|
| Gating strategy | A flow cytometer assessed the cell apoptosis after 48 h of transfection. Following the instructions of Annexin-V-FITC Cell Apoptosis Detection Kit (CA1020, Beijing Solarbio Science & Technology Co., Ltd., Beijing, China), Annexin-V-FITC, PI, and HEPES buffer were prepared into Annexin-V-FITC/PI dye solution at a ratio of 1:2:50. A total of 1 × 106 cells were resuspended per 100 μL dye solution. |

☒ Tick this box to confirm that a figure exemplifying the gating strategy is provided in the Supplementary Information.
